# Imaging sub-diffuse optical properties of cancerous and normal skin tissue using machine learning-aided spatial frequency domain imaging

**DOI:** 10.1117/1.JBO.26.9.096007

**Published:** 2021-09-23

**Authors:** Andrew C. Stier, Will Goth, Aislinn Hurley, Treshayla Brown, Xu Feng, Yao Zhang, Fabiana C. P. S. Lopes, Katherine R. Sebastian, Pengyu Ren, Matthew C. Fox, Jason S. Reichenberg, Mia K. Markey, James W. Tunnell

**Affiliations:** a The University of Texas at Austin, Department of Electrical and Computer Engineering, Austin, Texas, United States; b The University of Texas at Austin, Department of Biomedical Engineering, Austin, Texas, United States; c The University of Texas at Austin, Dell Medical School, Department of Internal Medicine, Austin, Texas, United States; d The University of Texas MD Anderson Cancer Center, Imaging Physics Residency Program, Houston, Texas, United States

**Keywords:** light scattering, optical properties, tissue optics, machine learning, artificial neural network

## Abstract

**Significance:** Sub-diffuse optical properties may serve as useful cancer biomarkers, and wide-field heatmaps of these properties could aid physicians in identifying cancerous tissue. Sub-diffuse spatial frequency domain imaging (sd-SFDI) can reveal such wide-field maps, but the current time cost of experimentally validated methods for rendering these heatmaps precludes this technology from potential real-time applications.

**Aim**: Our study renders heatmaps of sub-diffuse optical properties from experimental sd-SFDI images in real time and reports these properties for cancerous and normal skin tissue subtypes.

**Approach:** A phase function sampling method was used to simulate sd-SFDI spectra over a wide range of optical properties. A machine learning model trained on these simulations and tested on tissue phantoms was used to render sub-diffuse optical property heatmaps from sd-SFDI images of cancerous and normal skin tissue.

**Results:** The model accurately rendered heatmaps from experimental sd-SFDI images in real time. In addition, heatmaps of a small number of tissue samples are presented to inform hypotheses on sub-diffuse optical property differences across skin tissue subtypes.

**Conclusion:** These results bring the overall process of sd-SFDI a fundamental step closer to real-time speeds and set a foundation for future real-time medical applications of sd-SFDI such as image guided surgery.

## 1 Introduction

Light transport through biological tissue has been shown to be sensitive to microstructural composition, including the orientation of cells, the constituents of the intra- and extracellular matrix, and the ratio of cell sizes.^1^ This behavior can be characterized using optical properties such as the reduced scattering coefficient ($\mu_{s}^{\prime }$), absorption coefficient ($\mu_{a}$), and the phase function backscatter parameter (γ).^2^ These optical properties have been found to be useful in many medical applications such as detecting burn wound severity,^3^ monitoring blood occlusions,^4^^,^^5^ and aiding in cancer diagnostics.^6^^–^^9^

Methods to measure such optical properties have been developed and improved. In fiber optic based approaches, photons are injected at one location, and the remitted photons are detected at short distances away and analyzed to determine the optical properties.^10^ Although small fiber optics set-ups are effective, these point-based techniques have limited use in wide-field applications. To solve this issue, spatial frequency domain imaging (SFDI) has been developed, in which structured light is projected onto tissue and the light reflected is measured from the entire surface at once.^11^ Using sinusoidal light patterns of various spatial frequencies/phases and demodulating the reflected intensity measured at each frequency, SFDI can produce a demodulated intensity spectrum M(f) for each pixel of the imaged tissue. Calibrating these spectra yields a reflectance spectrum R(f) for each pixel. Optical property heatmaps can then be rendered from this grid of reflectance spectra.^11^^–^^13^

Recently, efforts have been made to advance the speed of SFDI up to real time, i.e., one frame per second,^14^ which involves increasing the speed of both the measurement of SFDI images and the rendering of optical property heatmaps from these images. Many methods have been developed which can significantly reduce the acquisition time needed for capturing wide-field SFDI images. These include methods which capture images at multiple wavelengths simultaneously^15^^,^^16^ as well as methods which reduce the number of SFDI snapshots needed to form an image.^14^^,^^17^ There have likewise been many advancements in increasing the speed of processing these measurements and rendering optical property heatmaps. These methods include optimized look-up tables,^18^ random forest regressors,^19^ and deep learning networks.^4^^,^^20^^–^^22^

These advancements, while impressive, have only been implemented for diffuse scattering reflectance. As such, they cannot be used to find the phase function backscatter parameter γ, a parameter which has been shown to be useful in discriminating between cancerous and non-cancerous breast tissue.^2^^,^^6^ This parameter is defined as $\gamma =(1-g_{1})/(1-g_{2})$, where $g_{1}$ and $g_{2}$ are the first and second Legendre moments of the phase function, respectively. ($g_{1}$ is also known as the anisotropy factor and is often written simply as g.)^23^ The parameter γ characterizes the probability that a photon will undergo large-angle scattering during a scattering event. Physiologically, research has shown that γ is related to the size-scale ratio of the particles in the tissue, which is the ratio of the tissue’s largest particles to its smallest particles.^24^

The diffuse photons measured by the aforementioned state-of-the-art SFDI methods are found in the 0 to $0.2\;{\mathrm{mm}}^{-1}$ spatial frequency range. These photons have traveled farther from their source of injection and deeper into the tissue (up to 24 mm deep in the absence of absorption)^25^ before reflecting out of the tissue. As such, the individual angles they scatter with as they traverse through the tissue are averaged out over the large area of tissue through which they travel. Because of this averaging, reflectance in the diffuse domain is virtually insensitive to γ.^26^ In order to capture information about γ, SFDI reflectance spectra must instead be measured and examined in the sub-diffuse domain, which typically comprises spatial frequencies of $0.5\;{\mathrm{mm}}^{-1}$ or greater for biological tissues.^26^ SFDI that is inclusive of the sub-diffuse spatial frequency range is known as sub-diffuse spatial frequency domain imaging (sd-SFDI). Reflected photons found in the sub-diffuse range have traveled shallower into the tissue (<0.4  mm)^25^ and undergone a smaller number of scattering events, making them more indicative of their initial scattering events. As such, they are more sensitive to γ and provide more information about the tissue at shallower depths. These photons are also sensitive to $\mu_{s}^{\prime }$, but insensitive to $\mu_{a}$.^26^ Recent work has explored capturing sd-SFDI images and analyzing the data to find γ and $\mu_{s}^{\prime }$,^2^^,^^6^^,^^26^ known as sub-diffuse optical properties. However, the great advancements that have been made to bring SFDI to real-time speeds in the diffuse domain have not been translated over to measuring and analyzing these spectra in the sub-diffuse domain.

**Fig. 1:**
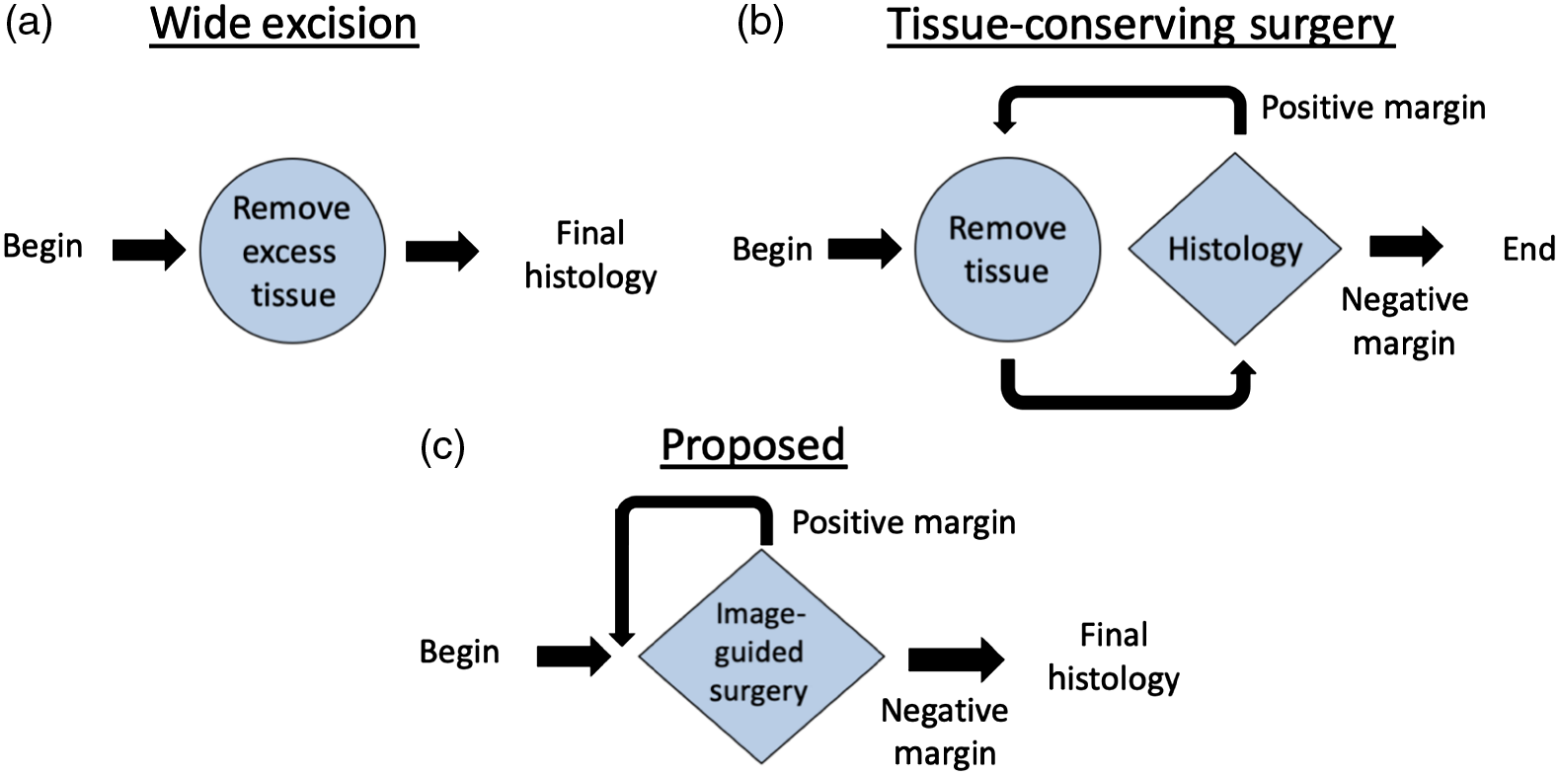
Flow diagram of existing and potential methods for removing cancerous tissue. All methods end in a final histological analysis to confirm all cancerous tissue has been removed. (a) In wide excision, a defined margin of normal tissue is removed along with the tumor in order to capture all cancerous tissue in one step. (b) In tissue-conserving surgery (e.g., Mohs micrographic surgery), small amounts of tissue are removed and then examined with histology repeatedly until all cancer is removed. (c) A potential method could use image guided surgery as a combined removal and examination step, resulting in a process that both captures all cancerous tissue in one step and mitigates normal tissue loss.

Improving the speed of sd-SFDI could open up a new opportunity in cancer treatment. Currently, there are two main methods by which surgeons excise cancerous tissue from a patient. The first is wide excision [Fig. 1(a)], a procedure in which the surgeon removes, in addition to the tumor, a defined margin of normal tissue around the tumor to ensure that all cancerous tissue is captured. At the end of this procedure, the removed tissue undergoes histological analysis to confirm negative margins.^27^ The other method is tissue-conserving surgery [Fig. 1(b)], e.g., Mohs micrographic surgery. During this process, a small amount of tissue is removed, and the topmost surface of this resected tissue is analyzed histologically for negative margins. If cancer is present at the evaluated margins, the process is repeated until all of the cancerous tissue is removed and a negative margin is confirmed. Because of the repeated histological assessments required, this procedure is time and labor intensive.^28^^,^^29^ The advent of wide-field sd-SFDI that identifies quantitative cancer biomarkers could allow for an improved method: image guided surgery [Fig. 1(c)]. Image guidance could allow the surgeon to remove minimal amounts of tissue without the need for repeated histological analysis, combining the speed of wide excision with the mitigated normal tissue loss of tissue-conserving surgery.

In image guided surgery, resected tissue could be diagnosed with imaging tools in place of histology. Alternatively, the excision site itself can be examined if the analysis method does not require removal of the tissue. In both cases, the target area of examination would be the topmost layer of tissue. The sub-mm penetration depth of sd-SFDI makes this imaging modality an attractive option for this scenario. Additionally, sd-SFDI is high resolution (<1  mm), it has a wide field of view (>1  cm), and it is a slide-free, stain free method.^30^

Image-guided surgery with sd-SFDI would require real-time measurements. More specifically, sd-SFDI images would have to be acquired in real-time and optical property heatmaps would have to be rendered from the sd-SFDI images in real time.^21^ This paper focuses on the latter challenge. Previous experiments have demonstrated rendering tissue optical property heatmaps from sd-SFDI images using least-squares minimization on a semi-empirical equation. ^2^^,^^6^^,^^26^ The challenges to using this method are two-fold. Primarily, the method requires 15 min for a 300×300  pixel image.^26^ Even for scenarios that do not require real-time imaging, such a long rendering time could serve as a significant bottleneck for advancing the knowledge of sub-diffuse optical properties in biological tissue, since such studies in the diffuse domain typically entail large datasets and images much larger than 300×300  pixels.^20^^,^^21^ Secondarily, the semi-empirical equation was developed using data simulated with the modified-Henyey Greenstein phase function approximation,^26^ whose domain^31^ does not fully encompass the range of optical properties seen in biological tissue.^2^^,^^21^^,^^26^^,^^32^^–^^35^

Recent work by Naglič et al. demonstrated a deep learning method for rendering sub-diffuse optical property heatmaps from sd-SFDI images that is capable of real-time speeds.^36^^,^^37^ However, while this method was shown to be successful for simulated data, this method has never been calibrated for and applied to physical experimental data. Moreover, Naglič et al.’s model was trained on data simulated using the Gegenbauer kernel (GK) phase function approximation, whose domain^38^ also does not cover the full range of optical properties seen in biological tissue.^2^^,^^21^^,^^26^^,^^32^^–^^35^

Another issue with sd-SFDI is that information on sub-diffuse optical properties is limited, and its use in cancer diagnostics has only been studied for breast tissue.^2^^,^^6^ One avenue where sd-SFDI guided surgery could be particularly useful is in the field of skin cancer, where tissue-conserving surgery is often performed using Mohs micrographic surgery.^28^ Moreover, with extremely rare exceptions, skin cancer does not spread any other way but contiguously.^39^ Because of this, if 100% of the excision surface is analyzed histopathologically, such as is done in Mohs surgery,^29^ and no signs of cancer are found, no additional or deeper tissue analysis is needed to declare a negative margin.^28^^,^^29^ This is in contrast to wide excision for skin cancer, which requires 4 to 6 mm of additional tissue to be removed and examined in order to declare a negative margin.^40^ Not only does this advantage allow Mohs surgery to conserve more tissue, it also makes Mohs a prime candidate for use with sub-diffuse SFDI, as it makes Mohs amenable to the limited penetration depth of sd-SFDI. Previous research has shown that diffuse SFDI could be useful for discriminating between cancerous and normal skin tissue,^41^^–^^44^ but little is known about the sub-diffuse optical properties of these tissue types.

This project is the first demonstration of real-time rendering of sub-diffuse optical property heatmaps from experimentally acquired sd-SFDI data. Moreover, we report preliminary data on the sub-diffuse optical properties of skin tissue subtypes. This work lays the foundation for implementing real-time applications of sd-SFDI, such as image-guided surgery.

## 2 Materials and Methods

In order to demonstrate real-time rendering of sub-diffuse optical property maps from sd-SFDI images, we acquired sd-SFDI images of two types of samples: tissue-simulating phantoms and human skin tissue samples. We imaged these samples across three wavelengths using a custom-built SFDI system. For each sample and wavelength, we collected 2050×2048  pixel demodulated reflectance images at 26 separate spatial frequencies, resulting in a [2050×2048  pixels×26 spatial frequencies] image cube per sample per wavelength. We then developed a Monte Carlo model with novel phase function sampling and used it to generate a training and validation dataset of simulated [1×26 spatial frequencies] sd-SFDI reflectance spectra (R(f)). This can be thought of as simulating individual pixels of sd-SFDI image cubes and collecting them into datasets. We also simulated a reference phantom spectrum for calibration $R_{\mathrm{ref}}(f)$ for each wavelength. We used the training dataset to iteratively train an artificial neural network (ANN) to predict sub-diffuse optical properties from sd-SFDI measurements. The validation dataset was used to validate that the model’s accuracy was improving during training iterations as to avoid overfitting the model to the training dataset.

We also fit a semi-empirical equation, developed by Kanick et al.,^26^ for benchmark comparisons. We tested the accuracy and speed of our ANN using the phantom measurements and compared the ANN’s performance to that of Kanick’s least-squared-minimization technique.^26^ We then applied the model to cancerous skin tissue samples and visualized the differences in optical properties between cancerous skin tissue and other skin tissue subtypes.

### 2.1 Sd-SFDI Process

**Fig. 2:**
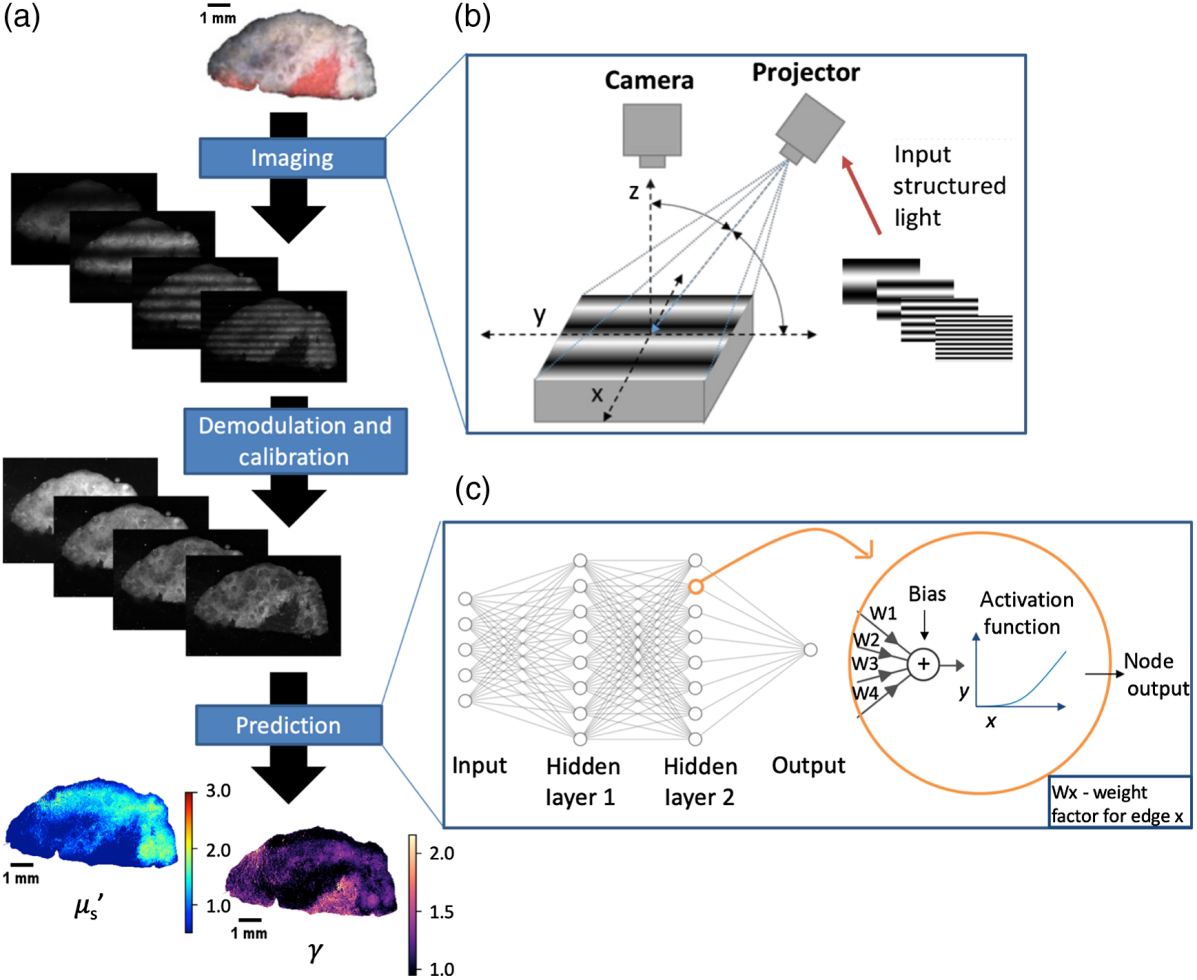
Sub-diffuse spatial frequency domain imaging method. (a) The full flow diagram for imaging a tissue sample and rendering a heatmap of its optical properties. This example uses four spatial frequencies. The tissue is imaged with the three-phase measurement method at various frequencies; the images are demodulated and calibrated for each frequency; and the resulting calibrated image cube is input into a machine learning model which calculates the optical properties. (b) To image the tissue, sinusoidal light patterns of varying spatial frequencies are projected onto the tissue, and the reflected light is measured with a camera. The axis of projection is orthogonal to the direction of the sine waves to decrease specular reflections and sensitivity to sample height variations. (c) A neural network similar to the example shown here is used to transform the reflectance spectra of each pixel into an optical property measurement. Each node’s inputs are aggregated in a weighted sum and passed through an activation function to yield the node’s output.

An overview of the sd-SFDI process can be seen in Fig. 2(a). To image the samples, we used a custom built SFDI system, diagramed in Fig. 2(b). A DLP Lightcrafter Evaluation Module digital micromirror device with built-in LEDs (Texas Instruments, Dallas, TX) was used to project sinusoidal light patterns of varying spatial frequencies and phases onto a sample. Based on experimental testing, a minimum of 12 pixels on the DMD was necessary to recreate accurate grayscale sinusoidal patterns. Due to the limited resolution of the DMD, a plano-convex singlet lens (f=15  cm) was placed in front of the DMD’s stock projection lens to decrease the size of the projected pixels, enabling the system to achieve spatial frequencies exceeding $1.3\;{\mathrm{mm}}^{-1}$. The system would otherwise be limited to only $∼0.4\;{\mathrm{mm}}^{-1}$ based on the minimum projection distance using the included projection optics. The sinusoidal patterns were projected onto the sample at an angle orthogonal to the patterns’ direction, and all samples were flat or flattened when imaged. This setup helped reduce specular reflections^45^ and made measurements less sensitive to variations in the samples’ height.^46^ By mitigating changes in the projected patterns’ spatial frequency as a function of distance from the projection axis, this orthogonal setup also made measurements less sensitive to the incidence angle and variations thereof over the length and width of the sample.^46^

A 5-megapixel monochrome CCD camera (piA2400-17gm Basler, Ahrensburg, Germany) was used to collect images of the light reflected from the sample.^47^ We employed the standard three-phase measurement method, by which measurements were acquired at three phases for each spatial frequency. These measurements were then demodulated.^11^ This process was repeated for 26 spatial frequencies (0.01, $0.025\;{\mathrm{mm}}^{-1}$, and 24 spatial frequencies evenly spaced between 0.05 and $1.25\;{\mathrm{mm}}^{-1}$) and 3 different wavelengths (450, 530, and 620 nm), resulting in an uncalibrated [2050×2048  pixels×26 spatial frequencies] image cube per wavelength, each pixel of each image cube containing a unique demodulated intensity spectrum, M(f). A calibration phantom was measured with this same method to find $M_{\mathrm{ref}}(f)$ per pixel per wavelength. Each pixel of each uncalibrated image cube was then calibrated using the equation $R(f)=M(f)/M_{\mathrm{ref}}(f)*R_{\mathrm{ref}}(f)$,^11^ resulting in a calibrated image cube for each wavelength.

The spatial frequencies used in this study include 6 frequencies in the diffuse domain ($f\leq 0.2\;{\mathrm{mm}}^{-1}$), 5 frequencies in the intermediate domain ($0.2\;{\mathrm{mm}}^{-1}<f<0.5\;{\mathrm{mm}}^{-1}$), and 15 spatial frequencies in the sub-diffuse domain ($f\geq 0.5\;{\mathrm{mm}}^{-1}$). The spatial frequencies in the diffuse domain were included as they improved estimation of $\mu_{s}^{\prime }$ (Supplement 1 in the Supplementary Material), which affects R(f) in both the sub-diffuse and diffuse domain.^2^ This also meant that optical property estimation had to account for possible variations in $\mu_{a}$, which affects R(f) in the diffuse domain.^11^ The upper bound of $1.25\;{\mathrm{mm}}^{-1}$ was chosen because higher frequencies showed a poor signal-to-noise ratio due to limitations in the dynamic range and sensitivity of the camera.^48^ Optionally, additional demodulation processing^11^ can be applied to the images to include the DC frequency, $f=0\;{\mathrm{mm}}^{-1}$, but validation tests showed no increase in prediction quality when including the DC frequency (data not shown), likely due to the frequencies very close to $0\;{\mathrm{mm}}^{-1}$ which are already included. As such, we did not include images at $f=0\;{\mathrm{mm}}^{-1}$ in our procedure.

An ANN similar to that developed by Naglič et al. was developed to predict optical properties from sd-SFDI reflectance spectra [Fig. 2(c)]. The model was a multi-layer perceptron with two hidden layers and an output layer,^37^ implemented with Keras and configured to work on OpenCL.^36^ The model, which has several weights that must be trained by fitting to observed data, ultimately behaves as an empirically trained analytical equation which maps the R(f) and $g_{1}$ of a pixel to an optical property value. As such, it computes very quickly. Two instantiations of this model were created and trained for $\mu_{s}^{\prime }$ and γ, respectively, allowing each model to more finely tune to its respective optical property (memory was not a considerable limitation). While one model with two outputs may have worked instead, using one model constrains the trained weights of the intermediate layers to have values that lead to accurate results for two outputs simultaneously, and only the final layer would be able to contain weights unique to each property. Using two distinct models rids us of this constraint. These models were used to render $\mu_{s}^{\prime }$ and γ heatmaps at all three wavelengths.

### 2.2 Generating Simulated Dataset and Training the Model

A simulated dataset was generated to train the ANN and the benchmark method’s semi-empirical equation. A diffuse Monte Carlo program developed by Hennessy et al.^49^ and Alerstam et al.^50^ was modified to include the parameter γ. This was achieved by developing a novel phase function sampling method. In this method, the phase function’s inverse CDF equation used for inverse transform sampling is replaced with an inverse CDF look-up table using methods similar to those introduced by Naglič et al.^32^ In order to allow for simulation across a wide range of the phase function parameters γ and $g_{1}$, our look-up table is generated with the modified Henyey-Greenstein^23^ (MHG) function when γ and $g_{1}$ fall within MHG’s valid range ($\gamma \leq 1+g_{1}$^31^) and is otherwise generated with the GK.^51^ We approximated GK’s valid range to be $\gamma >1+0.6g_{1}$ and $\gamma <3^{g_{1}}$^38^ (Supplement 1 in the Supplementary Material), so our method cannot sample phase functions with $\gamma \geq 3^{g_{1}}$. This is not a concerning limitation given the range of phase function parameters seen in our phantoms and in biological tissue.^2^^,^^21^^,^^26^^,^^32^^–^^35^ Our method uses a novel mapping which relates the γ and $g_{1}$ values to the GK parameters^38^ (Supplement 1 in the Supplementary Material) to calculate the GK parameters when using the GK phase function. It should be noted that the GK phase function has an analytical inverse CDF^52^ that can be used in tandem with our mapping in place of the inverse CDF look-up table, but using look-up tables for both phase functions allowed for more seamless switching between GK and MHG, the latter of which does not have an analytical inverse CDF.^32^

This hybrid MHG-GK phase function sampling method allows for simulations of R(f) across a wide range of phase function parameters encompassing the γ and $g_{1}$ values seen in both our phantoms and in biological tissue.^2^^,^^21^^,^^26^^,^^32^^–^^35^ The method’s ability to sample phase functions with a specified γ and $g_{1}$ combination, enabled through our GK mapping and MHG’s analytical relation to γ and $g_{1}$,^23^ allows for the simulation of a reference phantom’s spectrum $R_{\mathrm{ref}}(f)$. This is necessary for calibrating measurements of physical data so that the calibrated data can be input into models trained on simulated data.^11^

We verified that the phase function of the hybrid model was a good match for the phase function of the calibration phantom as calculated with Mie theory (Supplement 1B in the Supplementary Material). The modified Monte Carlo program with hybrid phase function sampling was then used to generate a training dataset, a validation dataset, and the calibration spectrum. Datasets were generated for combinations of the optical properties seen in Table 1, chosen to cover the range of optical properties seen in biological tissues^2^^,^^21^^,^^26^^,^^32^^–^^34^ as well as our tissue phantoms. A wide array of $\mu_{a}$ values was included to account for variations in $\mu_{a}$ values when estimating $\mu_{s}^{\prime }$. Table 1 also shows the optical properties of the phantoms whose measurements served as the experimental dataset for testing the model. The labels of the datasets, which the models would be trained to predict, were $\mu_{s}^{\prime }$ and γ. The features, which the models would use to make these predictions, comprised the reflectance values R(f) at the 26 spatial frequencies as well as the value of $g_{1}$. Table 1 shows the total number of samples used in each dataset. Combinations that fell outside the upper bound of the GK range (i.e., $\gamma \geq 3^{g_{1}}$) were discarded during simulation, shrinking the total number of samples in the training and validation dataset.

**Table 1:** Parameters used for training, validation, and experimental datasets. Formats of “[x,y]n”, signify n elements in the range of [x,y].

	$\mu_{s}^{\prime }$ (${\mathrm{mm}}^{-1}$)	γ	$g_{1}$	$\mu_{a}$ (${\mathrm{mm}}^{-1}$)	# of samples
Training	[0.5, 6.0] 25	[0.95, 2.20] 18	[0.10, 0.9] 4	[0.001, 0.50] 15	14625
Validation	[1.0, 5.5] 10	[0.96, 2.19] 5	[0.10, 0.9] 4	[0.084, 0.50] 6	330
Experimental	[1.1, 3.6] 15	[0.97, 2.17] 9	[0.07, 0.93] 8	0.001	15

The value of $g_{1}$ is assumed to be known ahead of time, allowing for its use as a feature. This is possible because $g_{1}$ is known for the phantoms and is assumed to be constant at 0.9^45^^,^^53^ for biological tissues. More specifically, the $g_{1}$ of soft mammalian tissue falls within the range of 0.8 to 0.95,^35^ and R(f) remains relatively invariant to changes in $g_{1}$ at $g_{1}$ values >0.8.^54^

We note that the training data did not entirely encompass the range of our testing data, which is an opportunity for future improvement with the model. However, the model still performed well when applied to testing data whose optical properties fell outside the range of the data the model was trained on.

The model was trained on the training dataset, and the validation dataset was used to determine the stopping point. More specifically, the model was set to train for 2000 epochs or until the mean squared error of the model on the validation dataset failed to improve for 100 epochs in a row.

While outside the scope of this study, we note that our sd-SFDI method could potentially also find $\mu_{a}$ values. The model already includes R(f) values at diffuse spatial frequencies in its inputs and is trained on simulations with a variety of $\mu_{a}$ values. However, the Monte Carlo simulations that the ANN was trained on used a semi-infinite geometry, where x and y are infinite in space, and z extends to negative infinity, which means that the ANN assumes the sample has infinite thickness. Although this assumption holds at sub-diffuse frequencies, where the penetration depth of the photons is shallow,^26^ the assumption does not hold when considering our tissue samples at diffuse frequencies. At diffuse frequencies, the penetration depth is in some cases deeper than the thickness of our tissue samples,^25^ which range from around 1 to 2 mm in thickness. Fortunately, the impact this has on our model’s $\mu_{s}^{\prime }$ calculations, which only partially relies on R(f) values at diffuse spatial frequencies, appears to be minimal, as shown in Supplement 2 in the Supplementary Material. However, the prediction of $\mu_{a}$ is likely to be adversely affected by low sample thickness, so further research would be required to examine and account for this impact before attempting to predict $\mu_{a}$. Alternatively, a future study could use thicker tissue samples.

### 2.3 Creating and Measuring Tissue-Mimicking Phantoms

Tissue-mimicking phantoms were created using polystyrene beads suspended in water (Polysciences Inc., Warrington, PA) with mean bead diameters of $d_{1}=0.0878\;\mu m$ (SD: 0.01  μm), $d_{2}=0.19\;\mu m$ (SD: 0.01  μm), and $d_{3}=0.99\;\mu m$ (SD: 0.03  μm). We prepared two dilutions of each bead distribution stock solution in distilled water: one to achieve a value of $\mu_{s}^{\prime }=2\;{\mathrm{mm}}^{-1}$ at 530 nm and the other to achieve a value of $\mu_{s}^{\prime }=2\;{\mathrm{mm}}^{-1}$ at 530 nm, as calculated using Mie theory.^55^ This resulted in six bead phantoms which covered γ values ranging from 0.97 to 2.17 and $\mu_{s}^{\prime }$ values ranging from 1.11 to $5.39\;{\mathrm{mm}}^{-1}$ to cover the ranges of values previously reported for various biological tissues.^2^^,^^21^^,^^26^^,^^32^^–^^34^ The optical properties of the phantoms at each wavelength were quantified using Mie Theory.^55^ All phantoms had negligible absorption (assumed to be $0.001\;{\mathrm{mm}}^{-1}$), as they were made with non-absorbing beads and distilled water. The six phantoms were measured at three wavelengths, resulting in eighteen total sd-SFDI image cubes.^47^^,^^56^ No cross polarization was used during sd-SFDI measurement as to avoid rejecting sub-diffuse photons.^57^

One of these six phantoms was measured before the rest of the phantoms to serve as a calibration reference, thus creating the reference spectrum $M_{\mathrm{ref}}(f)$ for each pixel and wavelength. When simulating this phantom to find $R_{\mathrm{ref}}(f)$ at each wavelength, $\mu_{a}$ was set to $0.001\;{\mathrm{mm}}^{-1}$ to represent negligible absorption, as a value of 0 caused problems with the simulator.

### 2.4 Measuring Skin Tissue Samples

Mohs surgery skin tissue samples were obtained in collaboration with the dermatologic surgery faculty at Dell Medical School. This study was approved and the informed consent requirement was waived by the Institutional Review Board at The University of Texas at Austin and the Seton Healthcare Family. We used four samples from three patients. Excision sites included the ear, the ankle, and the nose. The samples were frozen, the top layer of each sample was removed and processed using standard H&E staining methods, and digital microscopic images of the slides were captured and stitched.^58^ The thicknesses of the removed layers ranged roughly from 0.5 to 3 mm. Regions of interest delineating epidermis, dermis, and basal cell carcinoma (BCC) were outlined by a board certified dermatologist and fellowship trained Mohs surgeon (MCF) from Dell Medical School. The remainder of the sample was thawed and imaged using our custom SFDI set-up, using the same method and reference phantom that was used for measuring the phantoms. During imaging, tissue samples were positioned on the stage such that they fell within the same area the reference phantom was positioned in during imaging, but outside the area of the specular reflection that the reference phantom had. The tissue samples were flattened under a 1-mm glass slide to mitigate additional specular reflections. Marked H&E images of the tissues were co-registered with the SFDI heatmaps and used as a mask for each tissue subtype so that the optical properties of their pixels could be compared.

It is important to note that the co-registration between digital histology images and optical property heatmaps is not exact, as is likewise reported in the previous studies.^2^^,^^6^ The frozen sample from which the top layer is removed for histological analysis must be thawed for SFDI, resulting in slight shape changes to the sample. However, the heatmaps and H&E images are approximately the same shapes. The marked H&E images were manually rotated, scaled, and overlaid onto the heatmaps in MATLAB, and the markings were manually translated into the digital masks, keeping in mind other landmark features of the images to guide the making of the masks (Supplement 1C in the Supplementary Material).

Another limitation is that the histology image only captures the top-most layer, whereas the diffuse frequencies of the SFDI image are sensitive to deeper layers of the tissue.^56^ Moreover, since the top layer of the sample is removed for histology before the sample is imaged with sd-SFDI, this top layer itself is not captured in the sd-SFDI images.

### 2.5 Testing the Model

Average R(f) spectra were extracted from the 15 phantom sd-SFDI image cubes by averaging the spectra from select pixels of the image cubes at each wavelength (Supplement 1B in the Supplementary Material). To quantify the accuracy of the trained ANN model, the model was applied to these 15 averaged R(f) spectra along with their corresponding $g_{1}$ values to predict the optical properties of each phantom at each wavelength. The mean absolute relative error of the results for each optical property was calculated using the equation |ε¯|=1/n*∑in(yi−y^i)/yi.

The model was then applied to the SFDI image cubes of the phantoms to render $\mu_{s}^{\prime }$ and γ heatmaps of the phantoms. The SFDI image cubes of the skin tissue were then processed using the trained ANN to render heatmaps of $\mu_{s}^{\prime }$ and γ. The skin tissue was assumed to have an anisotropy factor of $g_{1}=0.9$.^45^ Software timing tools were used to measure the frame rate at which the model was able to render these heatmaps.

In tandem, $\mu_{s}^{\prime }$ and γ predictions were rendered using the semi-empirical non-linear fitting method first reported by Kanick et al.^26^

## 3 Results

### 3.1 Model Speed and Accuracy on Phantoms

**Fig. 3:**
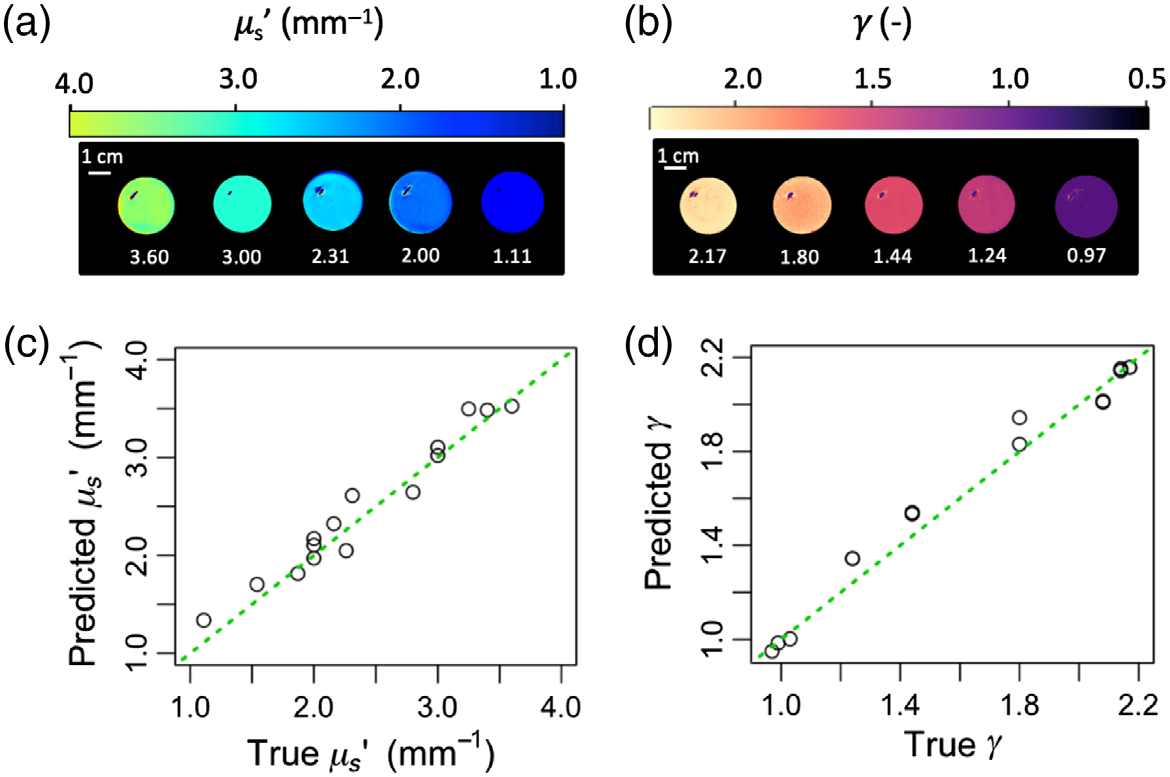
Performance of the neural network across five homogeneous phantoms with known optical properties and three wavelengths. Image cubes comprising 26 sd-SFDI images were transformed into optical property heatmaps for each phantom at each wavelength using the trained deep learning model, resulting in 15 heatmaps. (a), (b) Select $\mu_{s}^{\prime }$ heatmaps and select γ heatmaps, respectively, from the results, with the true value for the phantoms written in white underneath. Note these selections span multiple wavelengths and phantoms. The model accurately produces wide-field optical property heatmaps over the range of values tested. (c), (d) The accuracy of the model when run on an average spectrum from each of the phantom image cubes, where the x axis is the true optical property, the y axis is the predicted optical property, and the dotted green line represents unity. The mean absolute relative error was 6.8% for $\mu_{s}^{\prime }$ and 3.6% for γ.

The results of the model when tested on the experimental phantom spectra and image cubes are shown in Fig. 3. Looking at select sub-diffuse optical property heatmaps [Figs. 3(a) and 3(b)], we see that the model is able to correctly predict the optical properties of these phantoms, whose true values are shown in white text beneath the samples. Moreover, the model is able to make these predictions over a wide field and render optical property heatmaps from the sd-SFDI image cubes. One limitation to note, though, is that the heatmaps contain small artifacts from specular reflections in the liquid phantoms. This is likely due in part to not being able to use cross polarization. We avoided using this technique because it could reject sub-diffuse photons,^57^ but it is often used to filter out specular reflections.^45^ In order to mitigate the degradation of the inverse model’s performance caused by these artifacts, the specular reflections were avoided during all quantitative analysis of phantom heatmaps. This was done by selecting a section from each phantom that was free from specular reflections (Supplement 1B in the Supplementary Material). In future experiments, specular reflections may be reduced further by subtracting measurements of water in a deep well with dark walls from both the sample and reference phantom measurements.^2^^,^^45^

Quantifying the accuracies further, Figs. 3(c) and 3(d) show the predicted values plotted against the true values, with the solid line representing unity. The mean absolute relative errors of the $\mu_{s}^{\prime }$ predictions and γ predictions were 6.8% and 3.6%, respectively, which is within the range of error values previously reported in the literature.^2^^,^^6^^,^^26^ When run on simulated versions of these same phantom spectra, the model performed with an accuracy of $|\overset{¯}{\varepsilon }|=0.77\%$ for $\mu_{s}^{\prime }$ and $|\overset{¯}{\varepsilon }|=0.94\%$ for γ, on par with the accuracy that Naglič et al.’s model achieved over a narrower range of γ values.^36^

Comparing the speed of this method to the non-linear fit method previously used for sd-SFDI (Table 2), the ANN is able to render optical property heatmaps from 2050×2048  pixel image cubes in significantly less time. The frame rate of the non-linear fit method is only 0.0005 fps, i.e., a frame rendering time of 2000 s per frame. In contrast, the ANN run on the same device has a frame rate of 2.32 fps, i.e., a frame rendering time of 0.43 s per frame, which meets the requirement for real-time imaging.^14^ It is important to note that the ANN method is implemented with parallelization, allowing it to process multiple pixels simultaneously, while the non-linear fit method is not. In order to compare the speeds of the two methods independent of their parallelization, the speed of each process was examined when run on a single pixel. The non-linear fit method processed a single pixel with an average speed 0.013  s/pixel, while the ANN accomplished this in only 0.00187  s/pixel. Thus even without parallelization, the ANN still outperforms the non-linear fit method by an order of magnitude.

**Table 2:** Comparison of the frame rate for non-linear fit method versus artificial neural network.

Method	Frame rate (fps)	Frame rendering time (s/frame)	Pixel rendering time (s/pixel)
Non-linear fit	0.0005	2,000	0.013
Artificial neural network	2.32	0.43	0.00187

### 3.2 Tissue Type Comparison

**Fig. 4:**
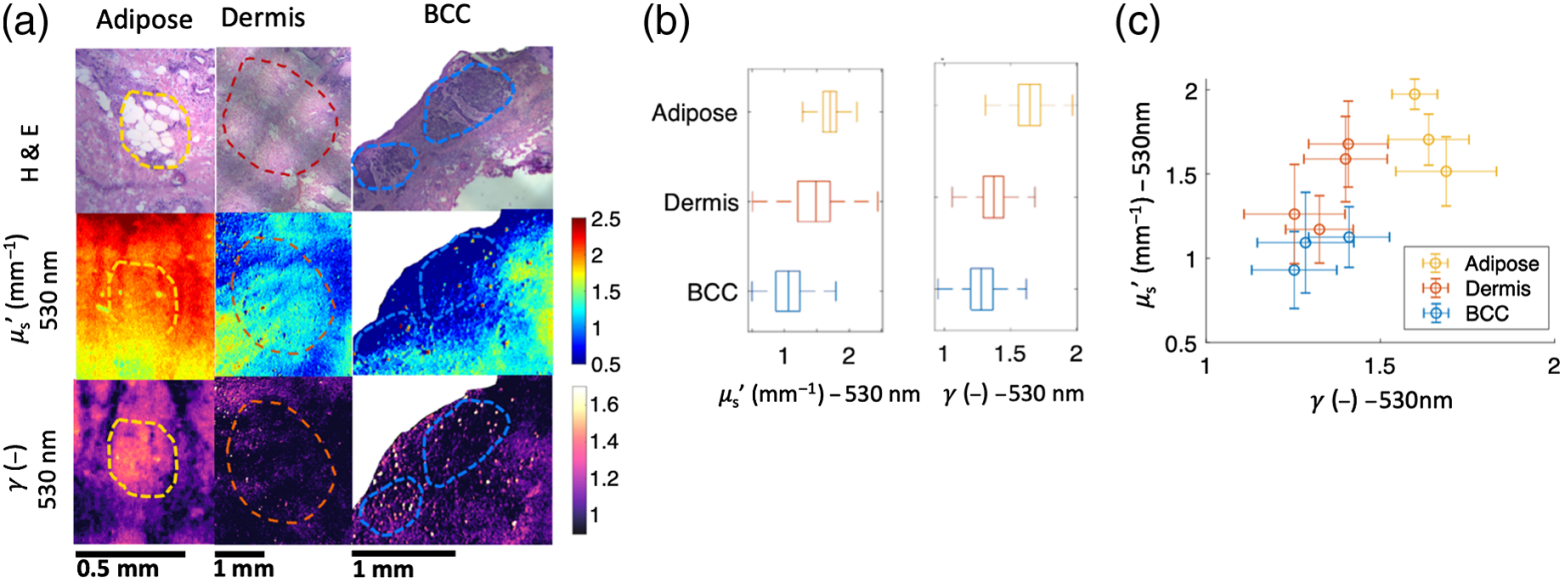
(a) Selected regions of the H&E images, along with corresponding regions of the $\mu_{s}^{\prime }$ and γ heatmaps at 530 nm, for each of the tissue subtypes. Areas of contrast which align with the marked regions of interest can be seen in the heatmaps. (b) Box and whisker plots of the optical properties of the pixels from each tissue subtype combined across all four tissue samples. BCC has lower $\mu_{s}^{\prime }$ than adipose and dermis, while adipose has higher γ than dermis and BCC. (c) Two-dimensional scatter plot of the mean values of the optical property for each sample’s tissue subtypes ± one standard deviation. The tissue subtypes fall into three distinct clusters, with γ separating adipose from BCC and $\mu_{s}^{\prime }$ separating Dermis from BCC.

Some highlighted results of the tissue experiments can be seen in Fig. 4. We focus on $\mu_{s}^{\prime }$ and γ at 530 nm in this figure because these two properties showed the greatest quantitative differences between the tissue subtypes as determined by visual inspection of all quantitative figures. A comprehensive figure with all results can be seen in Supplement 3 in the Supplementary Material. Figure 4(a) shows zoomed in regions of interest from the tissue samples for each tissue subtype. Dashed lines circle the regions of interest on H&E images, $\mu_{s}^{\prime }$ heatmaps, and γ heatmaps for each tissue type. Areas of contrast can be seen in the heatmaps that align with the boundaries of the tissue subtypes.

Adipose tissue exhibits high $\mu_{s}^{\prime }$, likely due to connective tissue septa which are particularly dense in subcutaneous fat in areas of the face and head,^41^ from which 3 of our 4 samples were excised. Adipose tissue also exhibits high γ, indicating that the large adipocytes result in a high ratio of the adipose tissue’s largest particles to smallest particles.^24^ Dermis exhibits a relatively high $\mu_{s}^{\prime }$ and a low γ, which may be caused by its collagen fibers and relatively small constituent particles.^59^ BCC tends to have a lower $\mu_{s}^{\prime }$ than normal skin tissue subtypes, echoing trends seen in the previous research.^41^^,^^43^^,^^44^ It also exhibits low γ values, which may be associated with its high density of small nuclei.^2^

To examine the sub-diffuse optical property differences between tissue subtypes, Fig. 4(b) shows box and whisker plots of the combined pixels from the tissue subtypes across all four tissue samples. In these data, while $\mu_{s}^{\prime }$ values of BCC show a fair amount of overlap with those of adipose and dermis, the inner quartiles of $\mu_{s}^{\prime }$ values for BCC show almost no overlap with those of adipose and dermis. Meanwhile, γ seems to separate adipose from dermis and BCC, as its inner quartiles show no overlap with those of dermis and BCC.

If these results prove to be consistent over a larger dataset, viewing them in the context of previous studies would suggest that γ is a more reliable feature than $\mu_{s}^{\prime }$ for discriminating between adipose and BCC. Salomatina et al. conducted a study using spatially resolved diffuse measurements with wavelengths ranging from 370 to 1600 nm. The study found that adipose tissue in skin excised from the face, neck, and scalp exhibited trends similar to our results, with $\mu_{s}^{\prime }$ higher in adipose tissue than in BCC. However, this study also found that adipose tissue in skin excised from the back exhibited low $\mu_{s}^{\prime }$ values, on par with those of BCC, due to its thin connective tissue.^41^ McClatchy et al., in a study on breast tissue using sd-SFDI at wavelengths of 658, 730, and 850 nm, found that adipose tissue from breast also exhibits low $\mu_{s}^{\prime }$ values. However, this tissue still exhibited high γ values similar to what is seen in our results.^2^ These results indicate that γ could be more consistently higher in BCC than in adipose tissue, whereas $\mu_{s}^{\prime }$ may not always be higher in BCC than in adipose tissue.

These findings suggest that using both of these properties in tandem could separate all three tissue subtypes from each other. To explore this idea further, Fig. 4(c) shows a two-dimensional scatter plot of the mean $\mu_{s}^{\prime }$ value versus the mean γ value from each tissue’s adipose, dermis, and BCC regions, ± one standard deviation. The optical properties of each tissue subtype form small clusters with overlap within standard deviations but separated means.

**Fig. 5:**
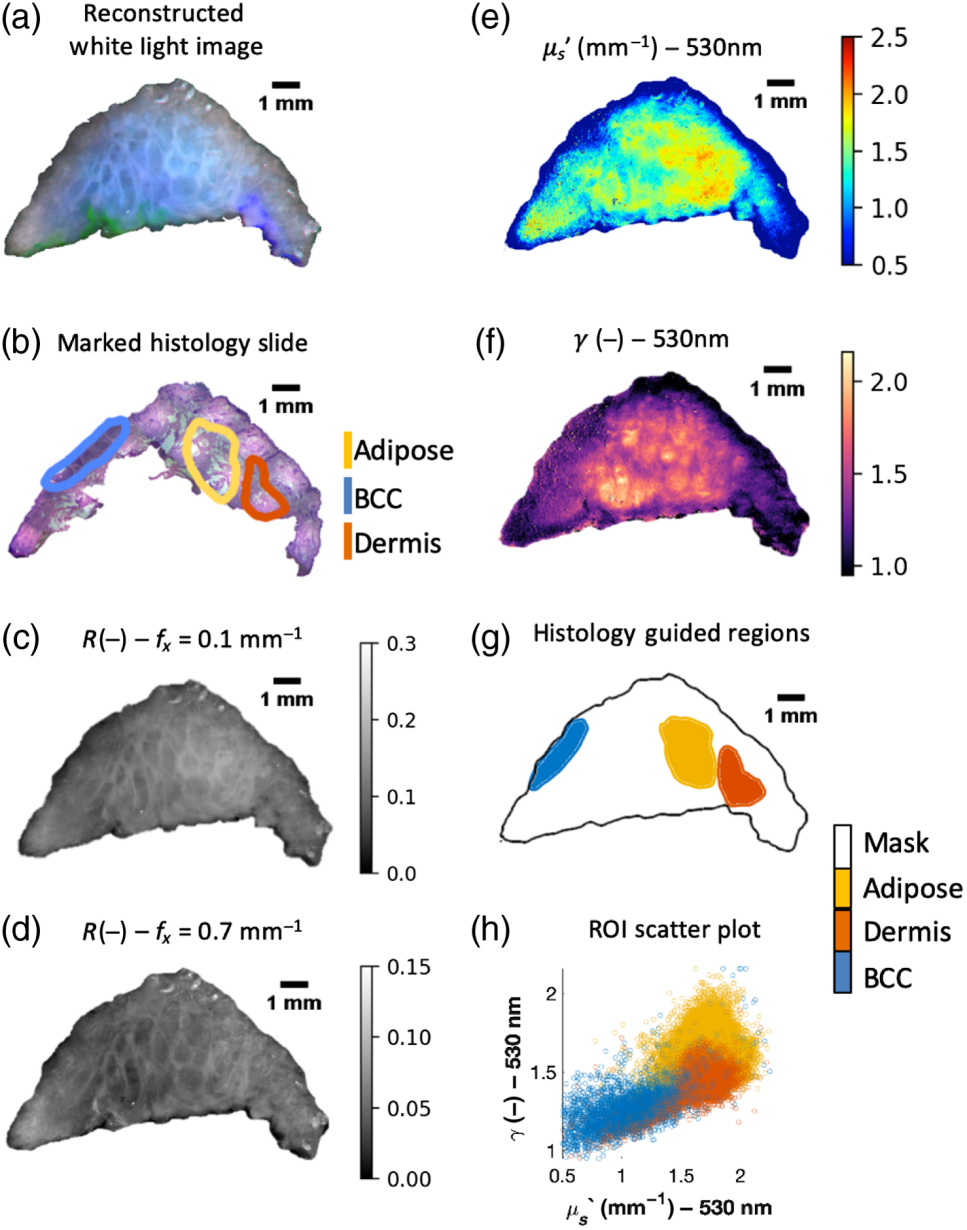
A representative tissue sample. (a) Reconstructed white light image of the sample. (b) Digital histology slide marked with regions of interest for the three skin tissue subtypes focused on for this study: adipose, dermis, and BCC. (c), (d) Demodulated and calibrated reflectance image for a diffuse and a sub-diffuse spatial frequency. Both images are taken at 530 nm. (e), (f) Optical property heatmaps of this sample for $\mu_{s}^{\prime }$ and γ, each at 530 nm. The images reveal structure not seen in the white light image. (g) Regions of interest from the marked histology slide translated onto the shape of the co-registered heatmaps. The regions of interest align with areas of contrast seen in the heatmap images. (h) $\mu_{s}^{\prime }$ versus γ scatter plot of all the pixels from these regions of interest. Pixels from subtypes fall into optical property clusters, albeit with overlap.

Figure 5 examines the results of a select tissue sample. Looking at a single-tissue sample over a wide range is more representative of a clinical scenario, in which a medical professional may use sub-diffuse imaging to help discriminate between tissue subtypes. Figure 5(a) shows a reconstructed white light image of this tissue sample, created using the demodulated DC intensity images at the red, green, and blue wavelengths as channels in an RGB image. This is not a perfect reconstruction, as the individual wavelengths were not white-balanced, but it serves to approximate a white light photo of the tissue. Figure 5(b) shows the marked H&E image of this sample. Figures 5(c) and 5(d) show examples of the sd-SFDI reflectance images at a diffuse spatial frequency ($f=0.1\;{\mathrm{mm}}^{-1}$) and a sub-diffuse frequency ($f=0.7\;{\mathrm{mm}}^{-1}$), respectively, taken at a wavelength of 530 nm. The microstructural variety present within the tissue sample is not apparent when examining the white light image or the reflectance maps. However, looking at the sub-diffuse optical property heatmaps of the sample in Figs. 5(e) and 5(f), we see that the sample contains a wide range of optical property values indicating vast differences in microstructure. Moreover, looking at the tissue subtype masks that the marked histology image was used to form [Fig. 5(g)], we see that some of the contrast seen in the heatmaps aligns with the tissue subtype regions of interest. Finally, looking at a two-dimensional scatter plot of $\mu_{s}^{\prime }$ versus γ for all pixels from these regions of interest [Fig. 5(h)], we see that the optical properties of the pixels from these subtypes fall into their own clusters, though these clusters do overlap.

In general, the heatmaps of the samples frequently showed contrast that corresponded roughly with the regions of interest, although this contrast did not go so far as to consistently create clear, obvious outlines of these regions of interest. The scatter plot shows clear trends between the optical properties of the tissue and the subtype of the tissue, supporting the hypothesis that sub-diffuse optical properties could be useful in identifying and discriminating between skin tissue subtypes.

## 4 Discussions and Conclusions

These results represent the first time that sub-diffuse optical property heatmaps have been rendered from experimental sd-SFDI images in real time. This advancement, if combined with real-time sd-SFDI acquisition, could bring wide-field measurement of sub-diffuse optical properties to real-time speeds relevant for clinical applications (≥1  fps).^14^ Real-time acquisition could be enabled by using state-of-the-art acquisition and demodulation techniques,^14^^,^^17^ which can be readily used for sd-SFDI, and using fewer spatial frequencies in the ANN. Work by Naglič et al. demonstrates that sub-diffuse optical property heatmaps can be accurately rendered with ANNs using as few as five spatial frequencies: (0.0, 0.1, 0.2, 0.4, and $0.8\;{\mathrm{mm}}^{-1}$).^36^

This heatmap rendering method can also be used for other applications outside of cancer detection, such as measuring burn wound severity^3^ or agriculture applications such as detecting subsurface bruising in apples.^22^^,^^60^ While the range of properties for which the model can produce accurate predictions is limited by the range of properties it is trained on, the model can easily be retrained on new datasets generated with the sd-SFDI Monte Carlo algorithm for any possible applications requiring a wider range of optical properties.

Our unique GK parameter mapping and combined MHG-GK phase function sampling method allow for targeted simulation of sub-diffuse reflectance spectra over a wider range of γ and $g_{1}$ values than previously possible. This sampling method enabled the application of the ANN to physical experimental sd-SFDI measurements. However, the method could also be used to simulate sub-diffuse spectra for other purposes, such as improving sub-diffuse optical property estimation from spatially resolved reflectance measurements.^61^

Examination of the sub-diffuse optical properties of the tissue samples revealed intriguing differences between the properties of the cancerous skin tissue regions and those of the normal tissue regions explored in this study. Moreover, the observed trends are congruent with the limited existing information on sub-diffuse optical properties of biological tissue in general as well as on skin tissue specifically.^2^^,^^26^^,^^41^^–^^44^ These preliminary data are exciting, but measurements of a large number of tissue samples from several different subjects are needed to identify differences in the sub-diffuse optical properties of skin tissue subtypes that are both statistically and clinically significant.

Future studies can also expand this work to other skin tissue subtypes, such as epidermis and hair follicles.^41^ Moreover, this work can be expanded to additional tissue types, with some caveats. Skin cancer is an ideal candidate for this work. There is already a well-established and widely used tissue-conserving surgery method, Mohs, in which examination of surface margins is standard, and the existence of remaining cancer can be confirmed or denied by examining the topmost layer alone.^28^^,^^29^ However, the limited penetration depth of sd-SFDI is less applicable to cancers for which examination of the surface margin alone is insufficient to confirm negative margins, such as breast cancer. Breast cancer surgery requires examination of deeper tissue (i.e., up to 2 mm below the tissue surface for ductal carcinoma *in situ*) to obtain clinically accepted margin clearances.^62^ Additionally, this work may be more difficult to incorporate for cancers for which tissue-conserving surgery is generally not considered a gold standard, such as with testicular cancer.^63^

There are several other potential future studies which could be very useful. One would be to run a similar experiment using different wavelengths of light. This study used wavelengths in the visible range. These wavelengths penetrate tissue less deeply,^43^ making them useful for taking the shallow measurements needed to examine surface margins during image guided surgery. However, skin tissue subtypes have been shown to have better separation of $\mu_{s}^{\prime }$ at longer wavelengths,^41^ and there could exist a similar trend for γ. This possible trade-off would be worth exploring. Another future study which could improve these results further would be to incorporate profilometry correction, a technique which has already seen wide use in SFDI research.^21^^,^^46^ All of our sd-SFDI measurements were captured with the angle of projection oriented orthogonally to the direction of the sine waves. Taking additional measurements where the angle of incidence is instead oriented parallel to the direction of the sine waves provides measurements that are highly sensitive to changes in the height of the sample and can be used to automatically correct measurements to account for these changes.^46^ An additional reason to explore using sd-SFDI with multiple pattern orientations is images captured this way can be used to determine collagen fiber orientation,^64^ information which is difficult to determine at wide-scale with other imaging techniques.^65^ The structure of collagen fibers in skin tissue directly relates to the tissue’s subtype,^41^ and combining this information with the tissue’s sub-diffuse optical properties could result in a powerful diagnostic feature set.

A possible limitation that we did not study is whether or not the freezing and thawing of the samples has any impact on the results of the optical property measurements. All samples were processed the same way, so any consistent effects from this would be consistent across all samples. However, it is possible that imaging the samples before freezing would yield different values of the optical properties. This could be problematic if that difference is significant, as the proposed use case of image-guided surgery does not involve freezing and thawing the tissue. There is also the possibility that optical properties found when imaging tissue that has not been frozen and thawed could be more accurate, or, more importantly, more useful in discriminating cancerous tissue. For these reasons, it would be useful to conduct a study on the impact of freezing and thawing a sample before determining its optical properties.

This study demonstrates a fundamental step forward in measuring sub-diffuse optical properties across a wide-field in real time and has already led to further projects in real-time sd-SFDI.^66^ It also motivates further exploration into the potential of these properties in differentiating between cancerous and normal tissue subtypes. Altogether, it provides a foundation for the prospective goal of ultimately incorporating sd-SFDI into real-time medical applications, such as image-guided surgery.

## Code, Data, and Materials Availability

The code used to generate the sd-SFDI reflectance simulations has been made publicly available, free to use with citation of this publication, at the following Github repository: https://github.com/UTBMIL/gpumcml_bmil. All other code is hosted on the following private Github repository: https://github.com/UTBMIL/RFTROOP. The code for the ANN was built off of code published by Dr. Peter Naglič. Please contact Dr. Naglič directly for inquiries to using this code.^36^ Phantom data and simulation has been made publicly available through the Texas Data Repository at https://doi.org/10.18738/T8/BCKNMU, free to use with citation of this publication. Clinical data used in this work may be made available for access to specific parties through the Texas Data Repository under a Material Transfer Agreement at https://doi.org/10.18738/T8/BCKNMU.
