# Supplementary material for: Imaging sub-diffuse optical properties of cancerous and normal skin tissue using machine learning-aided spatial frequency domain imaging
Source: J Biomed Opt. 2021 Sep 23;26(9):096007. doi: 10.1117/1.JBO.26.9.096007 (PMC8459901; doi:10.1117/1.JBO.26.9.096007)
Supplement: Supplementary file 1 [file JBO_026_096007_SD001.pdf]

Imaging sub-diffuse Optical Properties of Cancerous and Normal Skin Tissue Using Machine Learning-Aided Spatial Frequency Domain Imaging

Andrew C. Stier,[a] Will Goth,[b] Aislinn Hurley,[b] Treshayla Brown,[b] Xu Feng,[b] Yao Zhang,[b] Fabiana C.P.S. Lopes,[c] Katherine R. Sebastian,[c] Pengyu Ren[b], Matthew C. Fox,[c] Jason S. Reichenberg,[c] Mia K. Markey,[b,d] James W. Tunnell[b,*]

[a]The University of Texas at Austin, Electrical and Computer Engineering, 2501 Speedway, Austin, TX 78712, United States

[b]The University of Texas at Austin, Biomedical Engineering, 107 W. Dean Keeton, Austin, Texas 78712, United States

[c]The University of Texas at Austin, Dell Medical School, Department of Internal Medicine, 1501 Red River St, Austin, TX 78712, United States

[d]The University of Texas MD Anderson Cancer Center, Imaging Physics, 1515 Holcombe Boulevard, Houston, Texas 77030, United States

1 EXPANDED MATERIALS AND METHODS

A. Custom Monte-Carlo Simulator

A custom Monte-Carlo simulator was developed to simulate reflectance spectra as a function of source-detector separation distances given an input set of optical properties of the medium. The optical properties considered were the reduced scattering coefficient (μ_s'), the absorption coefficient (μ_a), the anisotropy factor (g_1), and the phase function backscatter parameter (γ). An open source Monte Carlo simulator[50] with wrapper functions[49] was used as a starting point, but the program's use of the Henyey-Greenstein probability distribution function (**Eq. S1**) to model the phase function did not allow for variation of the γ parameter. The Henyey-Greenstein function is often used as it has an analytical solution to its inverse cumulative distribution function (CDF), making it easy to randomly sample via inverse transform sampling[67]. However, the function is only valid when $g_2 = g_1^2$ [23], meaning $\gamma = 1/(1 + g_1)$. This limitation is acceptable when working with diffuse SFDI, as the reflectance in the diffuse domain is invariant

to γ[26]. However, a phase function which can handle variation in γ is necessary to model sub-diffuse reflectance, which is dependent on γ[23].

$$p_{\mathrm{HG}}(\cos\theta|g_1) = \frac{1}{2}\frac{1-{g_1}^2}{(1+{g_1}^2-2g_1\cos\theta)^{3/2}} \ , \quad \text{(Eq. S1)}$$

Past solutions to this problem have been to replace the Henyey-Greenstein model with Gegenbauer Kernel (GK)[32,36,37,51] (**Eq. S2**).

$$p_{\mathrm{GK}}(\cos\theta|\alpha_{GK}, g_{GK}) = \frac{2\,\alpha_{\mathrm{GK}}\,g_{\mathrm{GK}}(1-g_{\mathrm{GK}}^2)^{2\,\alpha_{\mathrm{GK}}}}{[(1+g_{\mathrm{GK}})^{2\,\alpha_{\mathrm{GK}}}-(1-g_{\mathrm{GK}})^{2\,\alpha_{\mathrm{GK}}}](1+g_{\mathrm{GK}}^2-2\,g_{\mathrm{GK}}\cos\theta)^{(1+\alpha_{\mathrm{GK}})}} \ , \quad \text{(Eq. S2)}$$

While this model also has an analytical solution to its inverse CDF[53], there is no published analytical solution mapping this equation's parameters, α_{GK} and g_{GK}, to the simulator's input optical properties which affect the phase function, γ and g_1. To overcome this obstacle, we generated GK phase functions for a wide range of α_{GK} and g_{GK} combinations and calculated the resulting functions' γ and g_1 values. This was done by first computing g_1 and g_2 using the equations $g_1 = 2\pi\int_{\theta=0}^{\pi} p(\theta)\sin(\theta)\cos(\theta)\,d(\theta)$ and $g_2 = 2\pi\int_{\theta=0}^{\pi} p(\theta)\sin(\theta)\,(\frac{1}{2}(3\cos^2(\theta) - 1))d(\theta)$[68], using numerical approximations for the integrals with MATLAB's trapz function. These values were then used to calculate γ. We paired the α_{GK},g_{GK} combinations with their resulting γ, g_1 combinations in a reverse look-up table,thus creating a mapping from desired γ, g_1 values to the necessary GK parameters[38]. This allowed us to sample the GK phase function when running a simulation for a given γ and g_1. It should be noted that the actual values of γ and g_1 of the GK curve produced using these looked up GK parameters will vary slightly from the desired γ, g_1 values[38]. Since g_1 can be used during Monte Carlo simulations for calculations

other than sampling the phase function[49], it is important that these calculations use the actual g_1 values to produce valid results.

We also plotted the possible combinations of γ and g_1 that could be created (Figure S1 (a)). We found that the GK model could only produce valid phase functions when the optical properties fell into the approximate bounds of $\gamma > 1 + 0.6g_1$ and $\gamma < 3^{g_1}$, as marked by the blue lines in Figure S1 (a). This did not cover the range of values seen in biological tissue[2,21,26,32–35].

Another past solution for including γ into the Monte Carlo phase function has been to use the Modified Henyey-Greenstein (MHG) phase function (**Eq. S3**)[69]. This equation does not have an analytical CDF but does have an analytical mapping of its parameters, β and g_{MHG}, to the properties g_1 and g_2 (**Eq. S4, S5**)[23], and, by extension, γ. We developed and used a look-up table in place of an analytical CDF, as was done by Naglič *et al.*[32] However, this phase function model is only valid in cases where $\gamma < 1 + g_1$ [31]. A visual representation of the γ and g_1 combinations possible with this phase function are shown in Figure S1 (b), with the blue line representing the boundary. This also does not cover the range of values seen in biological tissue[2,21,26,32–35].

$$p_{\mathrm{MHG}}(\cos\theta|\beta,g_{MHG}) = \beta\, p_{\mathrm{HG}}(\cos\theta|g_{MHG}) + (1-\beta)\,\frac{3}{2}\cos^2\theta\,, \quad \text{(Eq. S3)}$$

$$g_{MHG} = \beta g_1, \quad \text{(Eq. S4)}$$

$$g_2 = \beta {g_1}^2 + \frac{2}{5}(1-\beta) \quad \text{(Eq. S5)}$$

However, the range covered by the MHG function is complementary to that covered by the GK phase function. We therefore used a case-based method which combined the two phase functions, sampling from the GK function when $\gamma > 1 + g_1$ and sampling from the MHG function when $\gamma \leq 1 + g_1$. This allowed us to simulate spectra for combinations of γ and g_1 where $\gamma <$

3^{g_1}, as seen in Figure S1(c), which did cover the range of values seen in biological tissue[2,21,26,32–35].

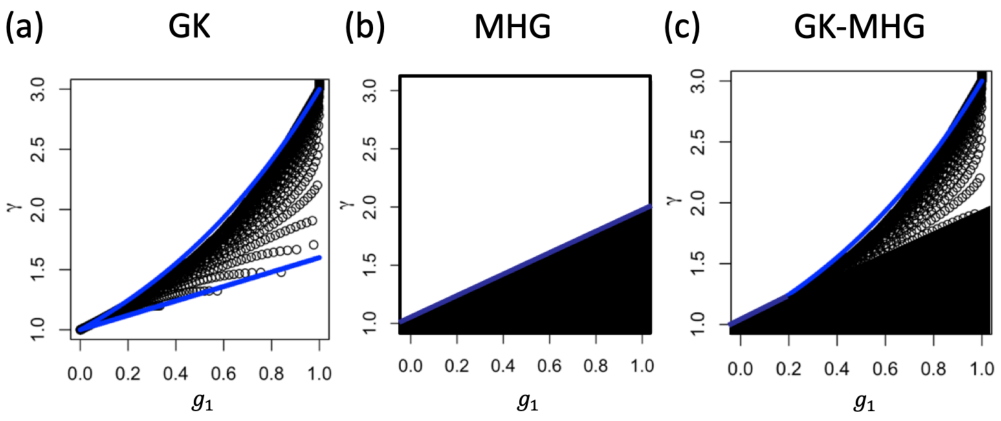

Figure S1. γ, g_1 combinations covered by each phase function. (a) The GK phase function has a visually approximated bound of $1 + 0.6g_1 < \gamma < 3^{g_1}$. (b) The MHG phase function is only valid in cases where $\gamma \leq 1 + g_1$. (c) The combined MHG/GK case-based method we present here is able to combine the complementary ranges of (a) and (b), allowing it to simulate all combinations of g_1 and γ such that $\gamma < 3^{g_1}$.

Reflectance vs. distance spectra were simulated using normal incidence and a radially symmetric detector with $1.35*10^6$ linearly distributed accumulator bins, each with a size of 10^{-5} cm, for a total simulation radius of 13.5 cm. These spectra were then converted to reflectance vs. spatial frequency spectra using the Hankel transform[11].

It should be noted that the incidence angle was low (<9°), but not 0°, so assuming normal incidence is an oversimplification. This is an area for possible improvements with our simulation algorithm. While not as robustly quantified for sub-diffuse measurements, a study by Tabassum *et al.*[70] showed that measurement of diffuse optical properties is robust to variations in angle up

to 9°. In our experiments, the results indicate that the simulator is still able to accurately simulate the reflectance spectra.

B. Creating and Measuring Tissue Simulating Phantoms

At the wavelengths used in this study (450 nm, 530 nm, and 620 nm), the beads had an index of refraction of 1.61, 1.60, and 1.59, respectively. Each phantom had a diameter of 2 cm and was 5 cm deep.

A MATLAB algorithm by Maetzler *et al*[55], which utilized Mie theory, was used to calculate the optical properties of phantoms with various microbead diameters and dilutions. These microbead parameters were chosen to yield phantoms with a wide range of optical property values. The theoretical optical properties for two of the phantoms at the three wavelengths used in this study can be seen in Figure S2(a) and (b). Figure S2(a) shows the properties for the reference phantom, which was one of the phantoms in which $\gamma \leq 1 + g_1$, while Figure S2(b) shows the optical properties of one of the phantoms in which $\gamma > 1 + g_1$. In the former scenario, γ decreases with wavelength, while in the latter, γ increases with wavelength.

A comparison of the MHG/GK hybrid phase function to the HG, MHG, and Mie phase functions for these two phantoms at a wavelength of 530 nm can be seen in Figure S2. Since for Figure S2 (c), g_1 and γ fall within the range of MHG, the MHG/GK phase function is the same as the MHG phase function. Conversely, in Figure S2 (d), g_1 and γ fall outside the range of MHG but within the range of GK, and the MHG/GK method samples the GK phase function. In both scenarios, the MHG/GK phase function is able to represent the Mie phase function well. Figure 2(d) also

shows oscillations in the Mie theory phase function, which makes the Mie phase function difficult to sample.

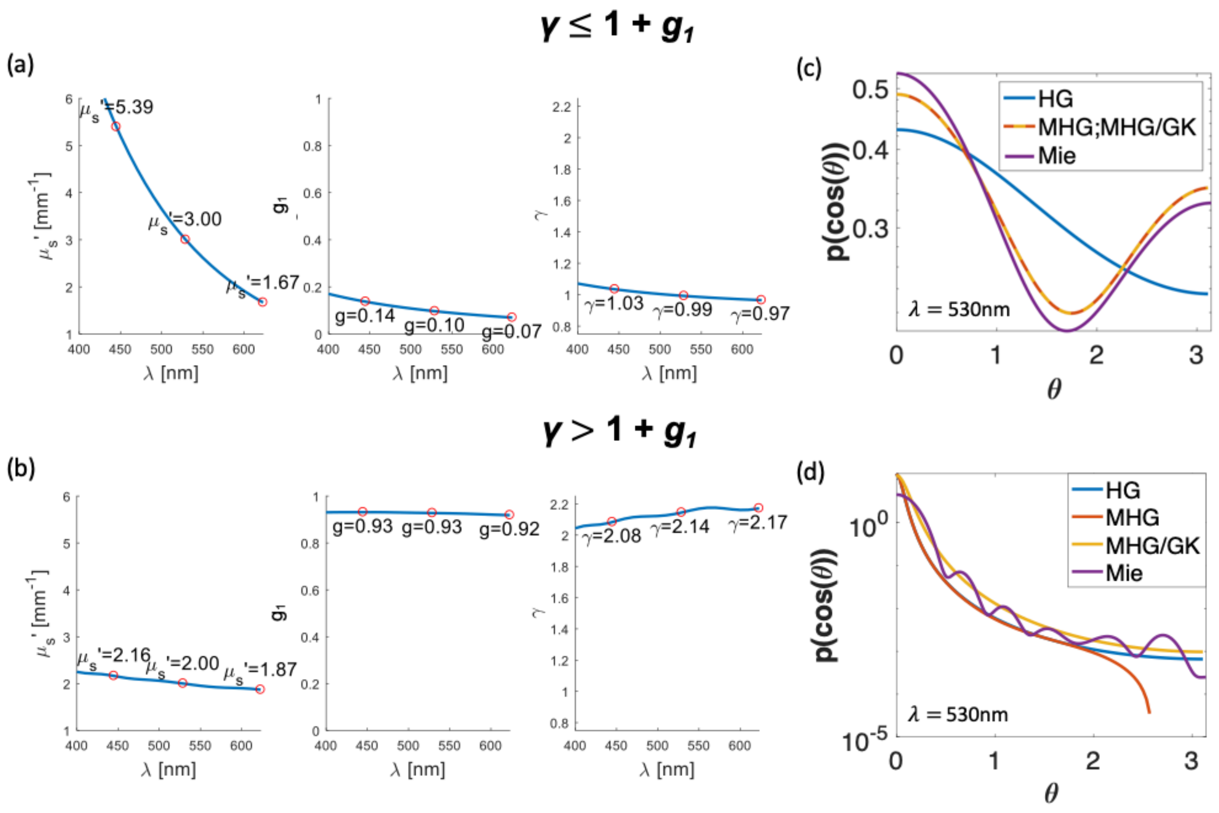

Figure S2. (a) and (b) Theoretical optical properties of the reference phantom and one of the experimental phantoms, calculated using Maetzler's Mie theory program. The values are highlighted with red circles and labels at 450 nm, 530 nm, and 620 nm. (c) and (d) Comparisons of HG, MHG, and MHG/GK phase functions to the Mie phase function for these phantoms at a wavelength of 530 nm. In (a), $g_1 = 0.1$ and $\gamma = 0.99$, while in (b) $g_1 = 0.93$ and $\gamma = 2.14$. Since the optical properties of (c) fall within the range of MHG, the MHG and MHG/GK phase function are the same, so their plots overlap directly. In this case, MHG/GK matches Mie better than HG and just as well as MHG. In (d), the optical properties fall outside of the range that MHG can properly model, and the MHG/HK method uses the GK phase function instead. In this case, MHG/GK matches Mie better than both HG and MHG.

The theoretical optical properties for the five phantoms used to create the experimental dataset can be seen in Figure S3. Note that this includes the phantom shown in Figure S2(b).

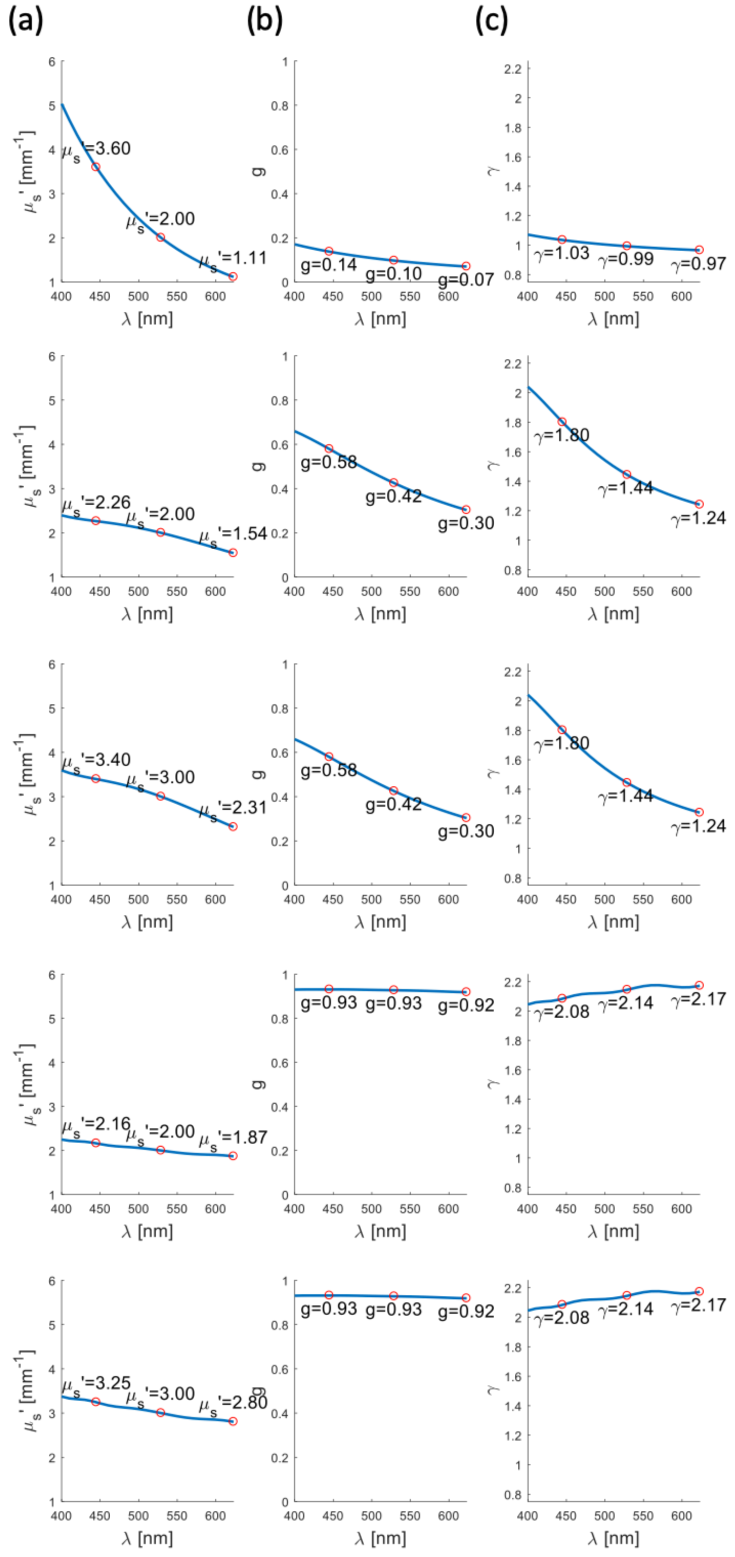

Figure S3. Theoretical (a) $\mu_s{}'$ values, (b) γ values and (c) g_1 values of the five phantoms used to form the experimental dataset plotted against wavelength, as calculated using Maetzler's Mie theory program. The values are highlighted with red circles and labels at 450 nm, 530 nm, and 620 nm.

Reconstructed white light images of the six polystyrene bead phantoms and their optical properties at 530 nm can be seen in Figure S4 (a). For each phantom, the spectra of a 200 x 200 square pixel area were averaged together to form one averaged spectrum. The colored squares indicate the area from which their spectra were averaged. The square area was chosen to avoid edge effects of the phantom as well as any image glare. An example of the averaged reflectance spectra from each of the phantoms at 530 nm can be seen in the dotted lines of Figure S4 (b), where the color of the dots matches the color of the square from which it came.

In order to provide more insight into the quality of the Monte Carlo model with MHG/GK phase function sampling, we include the simulated spectra of these phantoms at this wavelength, shown by the dashed lines in Figure S4 (b). The phantom represented by the red lines is the reference phantom, and the measured and simulated spectra match exactly, as expected. The other phantoms also line up very well, albeit not perfectly. This indicates that the calibration is not able

to perfectly match the simulated spectra with the measured spectra in every situation, and that the forward model is not able to account for all aspects of the experimental measurements.

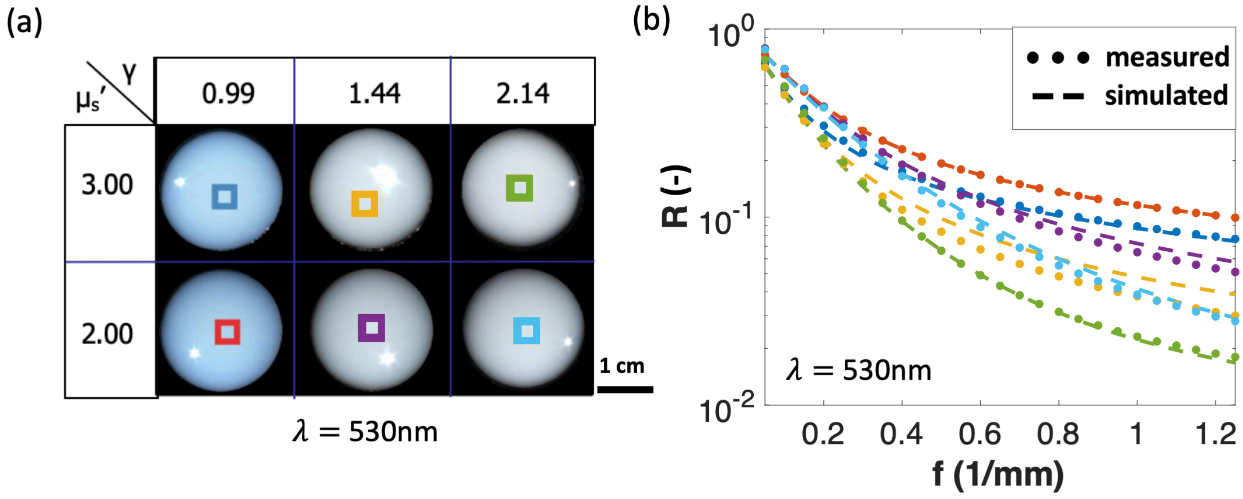

Figure S4. (a) Reconstructed white light images of the six phantoms used for this study, each with different μ_s' and γ value combinations. The phantoms look similar to the naked eye but are microstructurally very different from each other. The phantoms' μ_s' and γ values at 530 nm are shown here. The colored squares show the box areas that were averaged together for each phantom to create the respective phantom's average spectrum. (b) The resulting average spectra for these six phantoms at 530 nm, plotted in dotted lines, with each plot matching the color of the box from which it was created. The phantom marked in red was used as the reference phantom. Using the remaining five phantoms at three different wavelengths resulted in 15 experimental sd-SFDI reflectance spectra, each with a unique combination of μ_s' and γ. Plotted in dashed lines is the corresponding Monte Carlo simulations for each of these six phantoms at a wavelength of 530 nm. The matching of the reference phantom is trivial, but the other phantoms also match well.

C. Step-by-Step Co-registration

Figure S5 offers a step-by-step visualization of the co-registration process between H&E and sd-SFDI heatmaps, using one of the skin cancer tissue samples as an example. This example created a dermis mask for the tissue sample. After the optical property heatmap (Figure S5 (a)) and digital histology image are created, the histology image is marked by a board certified dermatologist to highlight the tissue subtype regions of interest (Figure S5 (b)). The histology image is then rotated, scaled, mirrored, and translated (using MATLAB's imrotate, imresize, flip,

and imtranslate, respectively) to co-register the histology image to the heatmap (Figure S5 (c)). Next, the regions of interest for the tissue subtype are traced (Figure S5 (d)), and the traced regions are converted into a subtype mask (Figure S5 (e)). This process is repeated for each tissue subtype and each tissue sample.

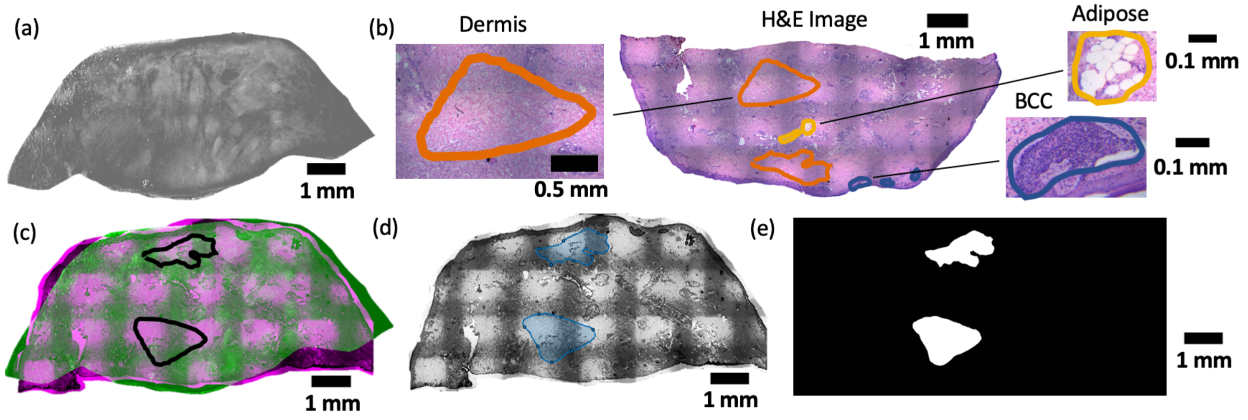

Figure S5. A step-by-step example showcasing how tissue samples are co-registered to histology images and tissue subtype masks are created. (a) An optical property heatmap of a tissue sample is produced using the sd-SFDI system and the ANN. Shown here is a γ heatmap. (b) The digital histology slide of the tissue sample is marked by a trained pathologist to outline sections of dermis, adipose, and BCC. Zoomed in sections of select marked areas are shown here. (c) The marked digital histology image is rotated, scaled, mirrored, and translated to co-register the image to the heat map. The co-registered images are shown here using MATLAB's 'falsecolor' display setting. For illustrative purposes, we have darkened the faint boundaries of the dermis sections, as this example created a dermis mask. (d) The regions of interest for the tissue subtype are traced on the co-registered histology image. In this example, the dermis is traced. (e) These tracings are converted into a binary mask.

The tissue types in the non-outlined areas of interest in the samples mostly include heterogenous tissue that cannot be considered strictly one subtype or another, as well as some homogenous areas of other tissue types that were not included in this study. These other tissue subtypes include sebaceous glands, hair follicles, and epidermis.

D. Selecting Sub-Diffuse Frequencies vs. Diffuse and Sub-Diffuse Frequencies

When selecting features for the model, we experimented with using only sub-diffuse frequencies, instead of using both diffuse and sub-diffuse frequencies. Figure S6 (a) shows the results of the

model's performance on the validation dataset when only using $R(f)$ at frequencies values above 0.5 mm^{-1}. Specifically, the frequencies used were 16 evenly spaced values between 0.5 mm^{-1} and 1.25 mm^{-1}, inclusively, with a spacing interval of .05 mm^{-1}. This resulted in a mean absolute relative error of 12.9% for μ_s' and 6.7% for γ.

Running this same experiment with diffuse and sub-diffuse frequencies (f = 0.01 mm^{-1}, 0.025 mm^{-1}, and 24 spatial frequencies evenly spaced between 0.05 and 1.2 mm^{-1}) results in a better model performance, as seen in Figure S6 (b). With $R(f)$ at these frequencies included in the model's feature set, the validation error for μ_s' falls to 4.7%. We also see a small improvement in the prediction of γ with an error of only 2.8%. With this in mind, we chose to use both the diffuse and sub-diffuse frequencies for our final model.

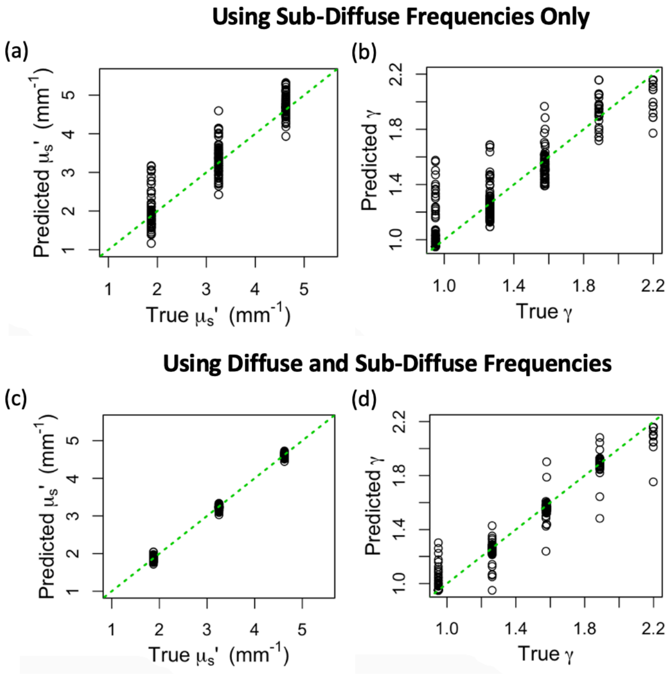

Figure S6. Results of the machine learning model on the validation dataset when the features used are g_1 and (a) $R(f)$ at 16 spatial frequencies evenly spaced from 0.5 to 1.2 mm^{-1}, and (b) $R(f)$ at f = 0.01 mm^{-1}, 0.025 mm^{-1}, and 24 spatial frequencies evenly spaced between 0.05 and 1.2 mm^{-1}. Using only sub-diffuse frequencies as seen in (a) results in a mean absolute relative error of 12.9% for μ_s' and 6.7% for γ, whereas including the diffuse frequencies as seen in (b) reduces this error to 4.7% for μ_s' and 2.8% for γ.

The sub-diffuse only model was not tested on the experimental dataset, which served as the test dataset. We did not want results on the test data to inform the feature selection, as that could result in overfitting to the test data.

D. Calculating Scatter Slope

Scatter slope is a property that has shown potential for identifying cancerous tissue in previous work[2]. The scatter slope, B, of the samples were found by fitting the equation $\mu_s'(\lambda) = \mu_s'(\lambda_0) * (\frac{\lambda}{\lambda_0})^{-B}$[2] to the μ_s' values of the sample at each of the three wavelengths, with the "reference" wavelength[71], λ_0, set equal to 620 nm.

2 ANALYSIS OF IMPACT OF THICKNESS

All of the training and validation data was simulated for mediums with infinite thicknesses. The tissue simulating phantoms, used for experimental test data, also had thicknesses large enough to be considered infinite. The tissue samples, however, varied in thicknesses ranging from roughly 1-2 mm, which is not thick enough to be considered infinite. This was expected to cause errors in the diffuse spatial frequency ranges, where the large absorbing layer (PDMS + India Ink) beneath the samples would introduce a high absorption coefficient and attenuate the diffuse spectra. The model was trained on a wide range of μ_a values in part to mitigate the impact of this effect. Absorption coefficient does not tend to impact the high spatial frequencies of the sub-diffuse regime[2], and the penetration depth of sub-diffuse photons is shallower[26], so we do not see impact from lower thicknesses in the sub-diffuse spatial frequencies. However, the model of this study uses reflectance at diffuse frequencies as part of its input, so we analyzed the impact of a low thickness on the model's accuracy.

In order to conduct this analysis, we edited our custom Monte Carlo model to include a second layer of infinite thickness and absorption, and we changed the original layer to have a thickness of only 1 mm, which is on the lower end of the thicknesses of the tissue samples used in this study. We simulated an experimental dataset with μ_s' values ranging from 1 – 4 mm^{-1}, γ values ranging from 0.96 – 2.19, μ_a of 0.001 mm^{-1}, and g_1 values of 0.9. We then ran our model to make predictions on this dataset. Plots of the results can be seen in Figure S7. In this scenario, we find a mean absolute relative error of $|\overline{\varepsilon}|$ = 8.4% for μ_s' and $|\overline{\varepsilon}|$ = 2.2% for γ.

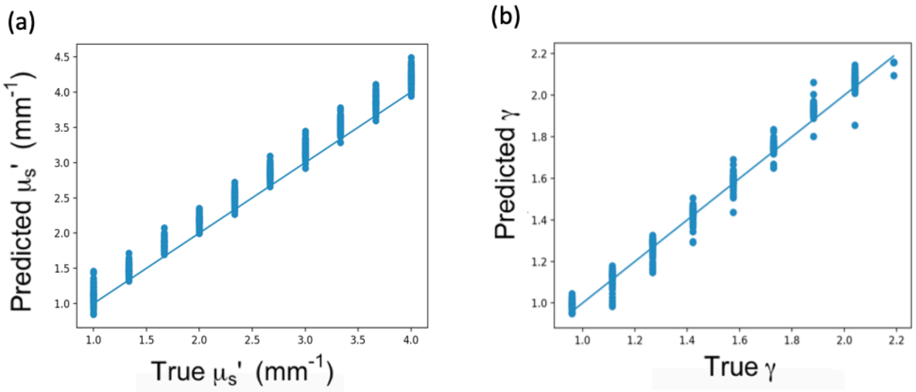

Figure S7. Performance results for the model when tested on data simulated to have a thickness of only 1mm. The y axis shows the value of the optical property predicted by the model, the x-axis shows the true value, and the straight line represents unity. (a) The μ_s' results show that the lower thickness introduces a bias to the μ_s' predictions, but the bias is low. The error in this situation increases to $|\overline{\varepsilon}|$ = 8.4%. (b) The γ results show that the lower thickness does not have a noticeable impact on the γ predictions, with the model achieving a low error of $|\overline{\varepsilon}|$ = 2.2%. These results demonstrate that our model is robust to the error introduced when using the model on tissue with finite thickness.

The reduced thickness of the media increases the error of the μ_s' predictions. As can be seen in Figure S7 (a), there is an offset bias to all of the μ_s' predictions. However, the error rate is still fairly low, within 10%. We see in Figure S7 (b) that there is no noticeable consistent bias introduced in the predictions for γ, and the error remains low. The parameter γ is primarily manifested in the reflectance at higher spatial frequencies, so it is as expected that the perturbations of spectra at lower spatial frequencies do not have a large effect on the model's ability to predict γ. Similarly, if we curtailed the 26 spatial frequencies used in this model to only

include the 15 spatial frequencies in the sub-diffuse domain, we would expect to see no difference between the model's bias on samples with different thicknesses, since the penetration depth of photons in the sub-diffuse is less than half a millimeter[25].

3 ADDITIONAL RESULTS

A. Phantom Results by Wavelength

Figure S8 shows the results of the ANN optical property measurements as well as the theoretical optical properties of the phantoms at each of the three wavelengths used in this study: 450 nm, 530 nm, and 620 nm. The agreement between the experimental and theoretical measurements does not appear to vary with wavelength.

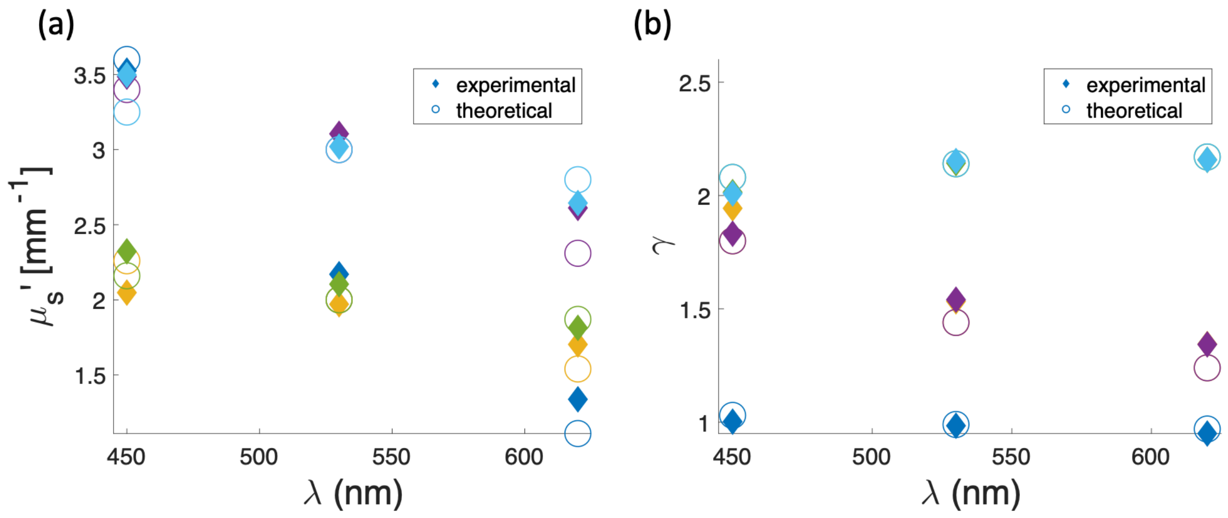

Figure S8. Experimentally measured and theoretically calculated (a) μ_s' values and (b) γ values of the tissue mimicking phantoms. Note: several theoretical values overlap. Phantoms are color coded. The agreement between the experimental and theoretical measurements remains fairly consistent at each wavelength.

B. Testing Scatter Slope Measurements

The phantoms created in this study were used to validate the measurement of μ_s' and γ. Unfortunately, the scatter slope power law assumption which has been used for biological tissue[2] does not hold for most of these phantoms, making them unsuitable for validating the

measurement of the scatter slope, *B*. However, two of the phantoms' μ_s' values do follow a power law. These two phantoms were used to examine the accuracy of measuring and calculating B. This analysis is limited by the small sample size. Another limitation is that one of these two phantoms is the reference phantom, so μ_s' predictions on this phantom may be overly optimistic.

The result of fitting the scatter slope equation to these phantoms can be seen in Figure S9. The reference phantom is in red, and the other phantom is in blue. The theoretical *B* values of the reference phantom and the other phantom were 3.66 and 3.04, respectively, while the *B* values found from the experimental μ_s' values of these phantoms were 3.67 and 3.69, respectively. These values resulted in a mean absolute relative error for of 8.9% for the measurement of *B*.

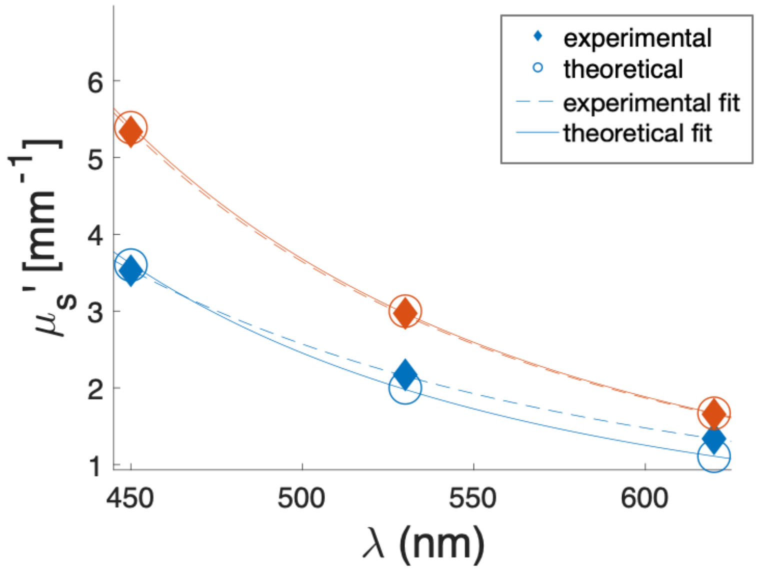

Figure S9. Results of fitting the scatter slope equation to the two of the six phantoms created in this study that follow a power law. This analysis is limited by the limited sample size as well as the fact that one of these phantoms is the reference phantom (shown in red). Calibrating the reference phantom is trivial, so μ_s' predictions and by extension *B* predictions on this phantom may be overly optimistic. Keeping these limitations in mind, the *B* values were measured with a mean absolute relative error of 8.9%. The experimental scatter slope fits can be seen with the dotted lines, and the theoretical fits can be seen in the solid lines.

C. Quantitative Non-Linear Fit Results

There are many limitations to comparing the accuracy of the ANN to the non-linear fit method, particularly because the latter was developed over a narrower range of optical properties ($\gamma \leq 1 + g_1$)[26,31] than the former. Keeping these limitations in mind, we provide an attempt to compare the quantitative results of the ANN to the non-linear fit method. The non-linear fit method was applied to the same experimental dataset as the ANN, and the mean absolute relative error was calculated using the values predicted with Mie theory for truth values, as was done for the ANN quantitative analysis. The results of this experiment can be seen in Figure S10. The method was able to find μ_s' with an error of 22.8% and γ with an error of 22.1%.

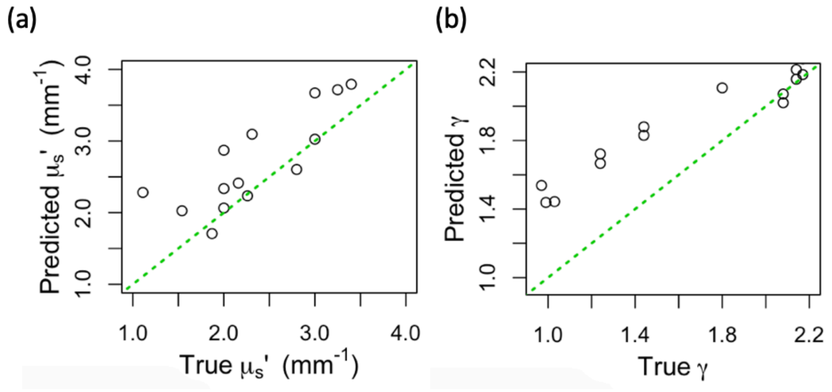

Figure S10. Accuracy of our implementation of the non-linear fit method when run on the average spectrum from each of the sd-SFDI phantom images, where the x-axis is the true optical property, the y-axis is the predicted optical property, and the dotted green line represents unity. Accuracy plots are shown for (a) μ_s' and (b) γ. The mean absolute relative error was 22.8% for μ_s' and 22.1% for γ.

The ANN therefore outperformed the non-linear fit method in both speed and accuracy. However, this result comes with many caveats. The implementation of fitting the non-linear equation to the measured spectra and finding the optical properties may not have matched that

used by the previous papers that have used this method[2,6,26]. For example, our implementation used the MATLAB function lsqnonlin to conduct the non-linear fit on the semi-empirical equation, but the functions used by previous papers is not listed. The non-linear fit method may also require better tuning of the parameters of the semi-empirical equation, [η, ζ_{1-4}], to account for differences between our experimental set-up and that of other labs, which fell outside the scope of this study. For this study we used the values reported by Kanick *et al.*[26] for ζ_{1-4} and manually chose a value of η that empirically matched the semi-empirical curves to the phantom data (η = 0.007).

At least two different versions of the non-linear fit method have been recorded. Kanick *et al.* applied the non-linear fit to only high frequency data ($f > 0.5$ mm^{-1}) using only the sub-diffuse semi-empirical equation[26], whereas McClatchy *et al.* applied the non-linear fit to the measurements at all frequencies using a hybrid sub-diffuse/diffuse semi-empirical equation[2]. Since the focus of this study is primarily on the ANN's gains in speed, we chose to compare against the former version as it was the faster of the two methods[2,26].

It should be noted that previous publications have reported accuracies with the non-linear fit method ranging from 10.2 - 23.8% for μ_s' and 3.9 – 6% for γ, but these tests were conducted over narrower ranges of optical property values, particularly with respect to γ (e.g. μ_s' = [1.1 – 2.5] mm^{-1}, γ = [1.3-1.6] for McClatchy *et al.*[2] and μ_s' = [0.4 – 1.8] mm^{-1}, γ = [1.4-1.75] for Kanick *et al.*[26] as opposed to this study's ranges of μ_s' = [1.1 – 3.6] mm^{-1}, γ = [0.97 – 2.17]).

D. Additional Samples and Heatmaps

Our model was used to render heatmaps of μ_s' and γ for four tissue samples at wavelengths of 450 nm, 530 nm, and 620 nm. In addition, the scatter slope, B, of each sample was calculated using the

measured μ_s' values at each of the three wavelengths. Figure S11 shows all samples used for tissue analysis, as well as all optical property heatmaps. Figure S11 (a) shows the marked histology images and the regions of interested translated onto the heatmap shapes. Figure S11 (c-d) shows the μ_s' and γ heatmaps, respectively of the samples at each wavelength. Figure S11 (d) shows the scatter slope heatmaps for these samples. Some areas of contrast can be seen around the tissue sub-type regions of interest for all optical property heatmaps. The starkest contrasts can be seen in the 450 nm and 530 nm.

The negative B values are concerning. Based on the size distributions of particles in tissue, B is typically positive, following the trend of the fractal dimension of the particles[72]. However, similar μ_s' vs. λ power law trends with negative B values can be seen in averaged SFDI skin tissue results which have been reported previously. These trends were specifically seen in patients whose Fitzpatrick scores ranged from III-V, signifying that they had darker skin tones[73]. While our samples did not come from patients with high Fitzpatrick scores, there could be a relation between the absorption of the tissue and the measured B value which would be worth exploring further in a future study which also measures absorption.

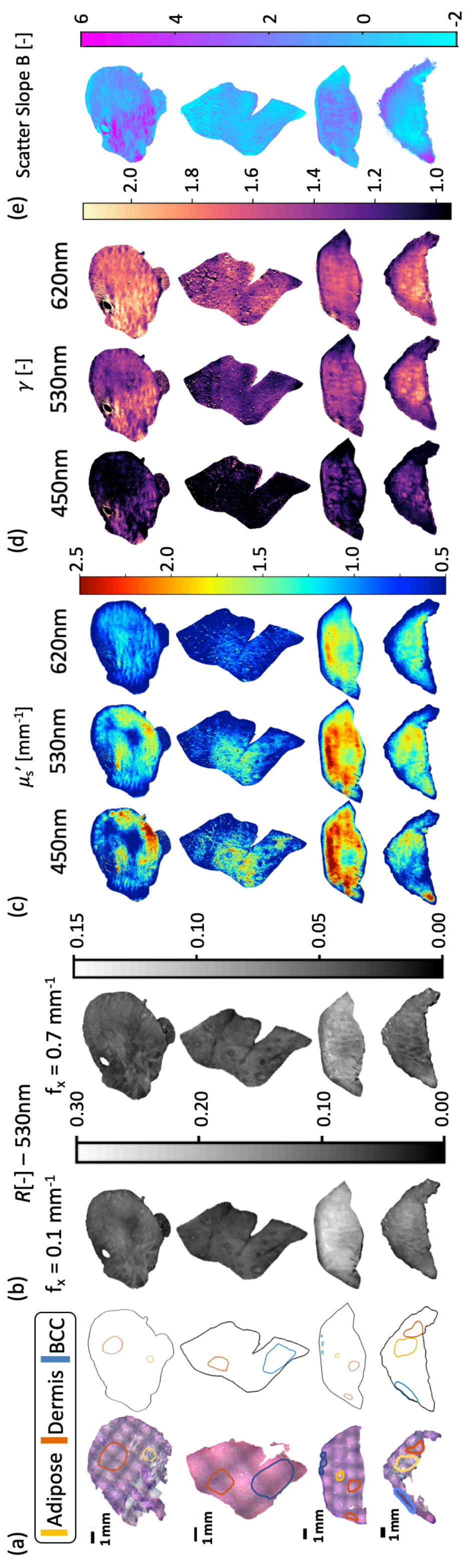

Figure S11. Additional samples and heatmaps. We show here the four skin tissue samples used in this study. (a) Marked digital histology slides and tissue sub-type heatmap masks. (b) Demodulated reflectance images at $f = 0.1$ mm^{-1} and 0.7 mm^{-1}. (c) μ_s' heatmaps at 450 nm, 530 nm, and 620 nm wavelengths. (d) γ heatmaps at 450 nm, 530 nm, and 620 nm wavelengths. (e) Scatter slope heatmaps. All heatmaps show a level of contrast that aligns with the boundaries of the different tissue subtypes.

E. Additional Data Analysis

Figure S12 (a-c) shows the box and whisker plots of all measured optical properties for the tissue subtypes. Many of the same trends in sub-diffuse optical properties can be seen between the different wavelengths, although the trends are most pronounced at 530 nm, suggesting that the contrasts seen in the 530 nm heatmaps have the best correlation with the tissue subtype regions. Adipose tends to have the highest γ value, while BCC tends to have the lowest μ_s' value. Figure S12 (d) shows a three-dimensional scatter plot B vs. μ_a vs. γ for all pixels from each tissue sub-type across all samples. We can see three distinct clusters, but the separation between clusters does not seem to change noticeably along the z-axis of scatter slope.

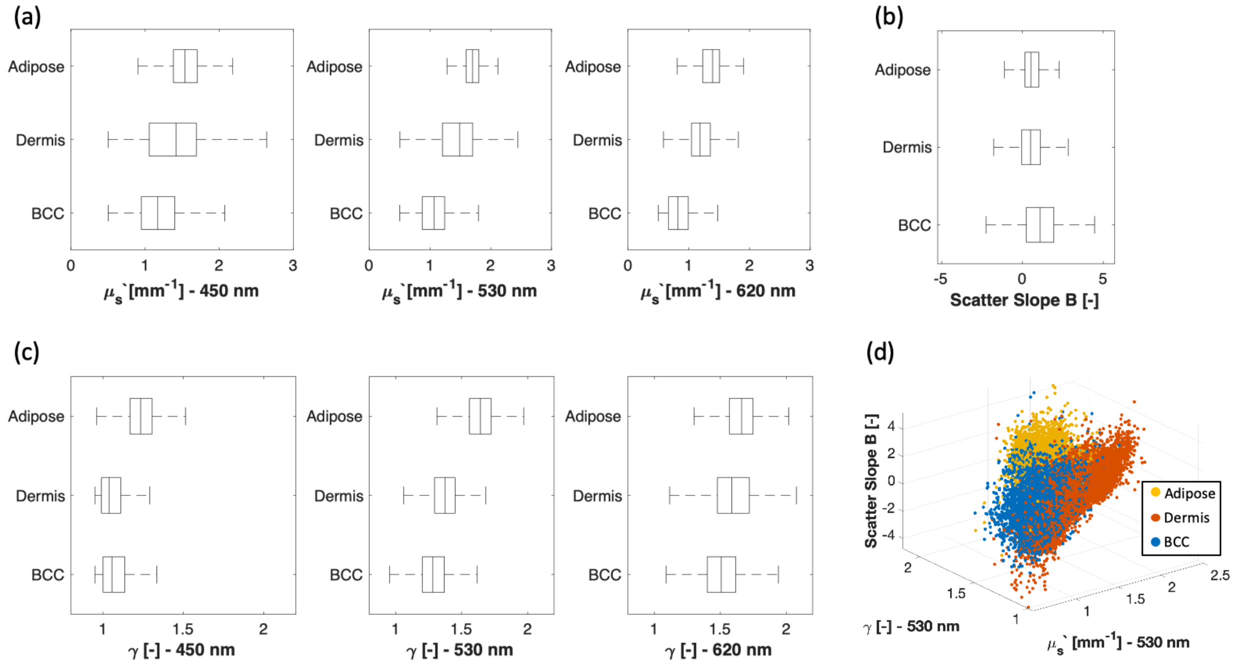

Figure S12. Additional data analysis of the optical properties of the different tissue subtypes, created with the pixels combined for each subtype from all four tissue samples. (a) Box and whisker plots for the μ_s' values of the tissue subtypes at 450 nm, 530 nm, and 620 nm. (b) Box and whisker plot for the scatter slope of each tissue subtype. (c) Box and whisker plots for the γ values of the tissue subtypes at 450 nm, 530 nm, and 620 nm. Similar trends between the tissue subtypes can be seen at each wavelength, with BCC generally having the lowest μ_s' and lowest γ of all the tissue subtypes. Conversely, it appears to have the highest scatter slope. (d) A 3-dimensional scatter plot of the scatter slope, γ, and μ_s' of all the pixels from each tissue-subtype collected across all samples. Distinct clusters with overlap can be seen.
